# Anti-Fn14-Conjugated Prussian Blue Nanoparticles as a Targeted Photothermal Therapy Agent for Glioblastoma

**DOI:** 10.3390/nano12152645

**Published:** 2022-08-01

**Authors:** Nicole F. Bonan, Debbie K. Ledezma, Matthew A. Tovar, Preethi B. Balakrishnan, Rohan Fernandes

**Affiliations:** 1 George Washington Cancer Center, George Washington University, Washington, DC 20052, USA; nbonan@gwmail.gwu.edu (N.F.B.); dkledezma@gwmail.gwu.edu (D.K.L.); mtovar@gwmail.gwu.edu (M.A.T.); preethibala18@gmail.com (P.B.B.); 2 Institute for Biomedical Sciences, George Washington University, Washington, DC 20052, USA; 3 School of Medicine and Health Sciences, George Washington University, Washington, DC 20052, USA; 4 Department of Medicine, George Washington University, Washington, DC 20052, USA

**Keywords:** photothermal therapy, prussian blue nanoparticles, aFn14 antibody, glioblastoma, targeted therapy, thermal therapy, immunogenic cell death

## Abstract

Prussian blue nanoparticles (PBNPs) are effective photothermal therapy (PTT) agents: they absorb near-infrared radiation and reemit it as heat via phonon-phonon relaxations that, in the presence of tumors, can induce thermal and immunogenic cell death. However, in the context of central nervous system (CNS) tumors, the off-target effects of PTT have the potential to result in injury to healthy CNS tissue. Motivated by this need for targeted PTT agents for CNS tumors, we present a PBNP formulation that targets fibroblast growth factor-inducible 14 (Fn14)-expressing glioblastoma cell lines. We conjugated an antibody targeting Fn14, a receptor abundantly expressed on many glioblastomas but near absent on healthy CNS tissue, to PBNPs (aFn14-PBNPs). We measured the attachment efficiency of aFn14 onto PBNPs, the size and stability of aFn14-PBNPs, and the ability of aFn14-PBNPs to induce thermal and immunogenic cell death and target and treat glioblastoma tumor cells in vitro. aFn14 remained stably conjugated to the PBNPs for at least 21 days. Further, PTT with aFn14-PBNPs induced thermal and immunogenic cell death in glioblastoma tumor cells. However, in a targeted treatment assay, PTT was only effective in killing glioblastoma tumor cells when using aFn14-PBNPs, not when using PBNPs alone. Our methodology is novel in its targeting moiety, tumor application, and combination with PTT. To the best of our knowledge, PBNPs have not been investigated as a targeted PTT agent in glioblastoma via conjugation to aFn14. Our results demonstrate a novel and effective method for delivering targeted PTT to aFn14-expressing tumor cells via aFn14 conjugation to PBNPs.

## 1. Introduction

Tumors of the central nervous system (CNS) are the most common tumor diagnosed in patients under the age of 14 and the 8th most common tumor diagnosed in patients over 40. CNS tumors account for a disproportionately higher rate of cancer-related mortality in the United States [1,2]. Glioblastoma (GBM) is histologically defined as a grade IV astrocytoma exhibiting incredible resistance to conventional treatment methods and an overall poor prognosis. The current point-of-care for GBM includes surgery, often followed by high-dose radiation therapy and administration of temozolomide (TMZ), a small molecule DNA-alkylating agent. GBM has a median survival length of 10–14 months even with the most aggressive treatment options [3,4,5,6,7,8]. Therefore, there is an urgent need for novel treatment options for GBM patients.

Photothermal therapy (PTT) mediated by light-absorbing and biocompatible nanoparticles that are activated by a laser is a promising treatment strategy for GBM. PTT has been extensively described by other groups and us as a means to achieve tumor control through both direct heat-based cytotoxicity, recruitment of the body’s endogenous immune system, and pro-immunogenic modulation of the tumor microenvironment [9,10,11,12,13]. Further, in the clinical setting, thermal therapies such as laser interstitial thermal therapy are safe and well tolerated in patients with non-accessible or recurrent GBM tumors [14]. This precedent indicates the potential application and enhanced efficacy of nanoparticle-based, targeted PTT for GBM.

In the context of GBM, several groups have described the use of PTT in vitro and in vivo [15]. The nanomaterials and nanoparticles investigated in these studies include metal-based, carbon-based, hybrid, and “other” nanoparticles that do not fit the aforementioned classifications [16,17,18,19,20,21,22]. The nanoparticles were either actively or passively targeted to GBM tumor cells or tumors to administer PTT. Overall, PTT induced significant cell death in both in vitro and in vivo models. The efficacy of these studies is summarized in a review by Bastiancich et. al. [15]. However, when tumor cells are heated using non-targeted nanoparticle-based PTT agents, the heating can be non-selective, with the potential for heating non-malignant tissue. To translate PTT to CNS tumors, non-selective heating should be limited to minimize the risk of off-target toxicities. Thus, a major step towards unlocking the potential of nanoparticle-based PTT for malignant CNS tumors is to engineer a robust, stable, and biocompatible tumor-specific nanotherapeutic modality with high on-target specificity without sacrificing direct tumor cytotoxicity.

To target nanoparticle-based PTT specifically to GBM tumor cells, we target the highly upregulated and GBM-specific receptor fibroblast growth factor-inducible 14 (Fn14). Fn14 is overexpressed on GBM tumors and is a cognate receptor for the tumor necrosis factor weak inducer of apoptosis (TWEAK) [23]. GBM is an aggressive tumor, often invading deep into the normal brain parenchyma, making it difficult for surgical resection and a major reason for tumor recurrence in treated patients. Fn14 receptor and its binding ligands have been shown to be significantly upregulated in poorly prognostic glioma compared to healthy neuronal or glial tissue and thus it represents a favorable candidate for developing targeted nanotherapeutics against GBM, mitigating off-target complications [22,23,24,25,26,27]. Fn14 also serves as a promising target not only for its abundance in GBM, but also its localization. Fn14 is expressed within both the tumor core and the invasive outer rim region of GBM, making it a potent target to treat all regions of this highly invasive tumor. [24,25] Furthermore, Fn14-TWEAK engagement leads to poor prognostic events, including proliferation, migration, and further invasion of GBM, which is often correlated with GBM’s resistance to chemotherapeutics [24,26,27,28]. Hence, engaging or blocking Fn14 expressed on the GBM tumor cells could make it unavailable for TWEAK binding and could be utilized to elicit antitumor effects. This suggests Fn14 could serve as a therapeutic target for targeted nanoparticle-based PTT.

Few groups have investigated the use of anti-Fn14 conjugation to nanoparticles. One group demonstrated that anti-Fn14 conjugation to gold nanoparticles acted as a TWEAK agonist, which would suggest activation of a more aggressive tumor cell phenotype [29]. This effect presents a strong argument for using PTT to ablate the tumor once the particles are delivered and bound to the surface of the GBM tumor cells. Another group demonstrated that aFn14 conjugation to carboxylate-modified polystyrene nanoparticles enhanced particle retention in on the surface of and within Fn14-expressing tumor cells [30], providing further rationale for Fn14-based targeting. However, while these studies show promise, the feasibility of translating them to clinic would be restricted by the limited clinical studies into the potential toxicities of gold nanoparticles and polystyrene nanoparticles [31,32]. Therefore, we used PBNPs conjugated with aFn14 as a nanotherapeutic with a potentially higher translational feasibility.

Prussian blue is an FDA-approved material used to treat radioactive poisoning when orally consumed as Radiogardase^®^ [33,34]. Additionally, PBNPs can be easily synthesized at large scales using low cost starting materials and is therefore amenable to clinical translation. We have previously described that, when irradiated by an 808-nm near infrared (NIR) laser, PBNPs facilitate photothermal energy conversion [35]. This energy conversion, mediated by electron-phonon coupling, followed by phonon-phonon relaxations in the setting of NIR radiation incident on the PBNP crystal lattice structure [9,36], has the capacity to produce tumor microenvironmental temperatures upwards of 80 °C, depending on the nanoparticle concentration and incident laser power, [9,10] triggering thermally induced cell death. Tumor cell death via this method is one of the most critical effects of PTT, as demonstrated by our group and others [37].

In addition to the safety offered by PBNPs, these particles also offer an immunological advantage. PTT can induce the release of endogenous immunoadjuvants such as damage-associated molecular patterns (DAMPs) from dying cells, indicative of a process known as immunogenic cell death (ICD) [38,39,40]. ICD can convert an immunologically “cold” tumor to a “hot” one, where released antigens can activate an immune response against the tumor. Our group and others have previously shown that PBNP-mediated PTT can induce ICD in pediatric neuroblastoma [9,10,41,42], melanoma [43], and triple-negative breast adenocarcinoma preclinical models, to name a few, and is being investigated in this study as a novel treatment option for GBM. ICD is not always induced by nanoparticle-mediated PTT, but the consistency with which our lab has observed PBNP-PTT-induced ICD makes PBNPs an attractive material.

The overall hypothesis in this study is that covalent conjugation of the Fn14 targeting antibody to the surface of the PBNP results in a stable nanotherapeutic (aFn14-PBNP) that can be utilized for targeted PTT without sacrificing the ability of the PBNPs to generate on-target heat-based cytotoxicity and ICD. We test this hypothesis by optimizing the synthesizing scheme for generating aFn14-PBNPs and assessing the critical quality attributes of the resulting nanotherapeutic including the attachment efficiency of aFn14 on PBNPs, the size distributions, surface charge, and UV-Vis-NIR spectra of aFn14-PBNPs. We then test the ability of the aFn14-PBNPs to elicit thermal and immunogenic cell death as well as effectively target and be retained on multiple human GBM tumor lines in vitro. Finally, in a targeted treatment assay, we test the ability of aFn14-PBNPs to target and treat GBM tumor lines with differential Fn14 expression compared to non-targeted PBNPs in vitro. Through our results, we seek to demonstrate that this novel nanoparticle-antibody conjugate provides a benefit over nanoparticles alone by retaining particles on aFn14-expressing tumor cells, thus enabling targeted PTT. These targeting studies with our aFn14-PBNP nanoformulation provide important proof-of-concept data to proceed with evaluating aFn14-PBNPs for PTT of GBM in vivo.

## 2. Materials and Methods

### 2.1. Synthesis of PBNPs

PBNPs were synthesized using a one-pot scheme as previously described [9,35,41,44]. Briefly, 20 mL of 10 mM aqueous FeCl_3_•6H_2_O (54.06 mg) (Millipore Sigma, Darmstadt, Germany) containing 5 mmol of citric acid (961 mg) (Millipore Sigma, Darmstadt, Germany) was added to an equal volume of 10 mM aqueous K_4_[Fe(CN)_6_]•3H_2_O (84.5 mg) (Millipore Sigma, Darmstadt, Germany) containing 5 mmol of citric acid, under vigorous stirring at 60 °C using a magnetic stirring hot plate. After heating and stirring for 60 s, the solution was allowed to cool to room temperature (RT) for 5 min under constant stirring. The 40 mL solution was then divided equally between two 50 mL tubes and PBNPs were then collected by adding equal volume of acetone (Millipore Sigma, Darmstadt, Germany) and 5 mL 5 M NaCl (Millipore Sigma, Darmstadt, Germany). The solutions were then centrifuged at 10,000 rpm for 15 min at RT. The centrifugation step with 20 mL MilliQ water, 20 mL acetone, and 5 mL 5M NaCl was repeated twice. The final PBNP pellet was sonicated (at 40% amplitude for 30 s using a microtip probe) in 10 mL MilliQ water to achieve colloidal resuspension. The PBNPs were stored under ambient conditions in DI water prior to further bioconjugation.

### 2.2. Synthesis of Bioconjugated aFn14-PBNP

Covalent synthesis of aFn14-PBNPs was carried out using 1-ethyl-3-(3-dimethylaminopropyl) carbodiimide (EDC) chemistry. To begin, 21.5 µL of the as synthesized PBNPs (23.21 mg/mL) was combined with 100 µL of EDC solution (2.2 mg/mL; Thermo Fisher Scientific, Waltham, MA, USA) and 100 µL of Sulfo-NHS solution (8.0 mg/mL; Millipore Sigma, Darmstadt, Germany) in 1 mL of MES buffer (0.1 M MES, 0.5 M NaCl, pH = 5; ACROS Organics, Geel, Belgium). The first crosslinking reaction occurred for 15 min at RT and was stopped via addition of 100 µL of 2-mercaptoethanol (8.9 mg/mL; Thermo Fisher Scientific, Waltham, MA, USA). PBNP crosslinked to Sulfo-NHS were then centrifuged at 22,000× *g* for 30 min using a table-top microcentrifuge unit at RT. Particles were resuspended in 1 mL MES buffer and sonicated using a microtip probe at 40% amplitude for 30 s to achieve a homogeneous colloidal solution. FITC-conjugated aFn14 antibody (Santa Cruz Biotechnology, Santa Cruz, CA, USA) was then added to a final concentration of 0.25 µg/mL, corresponding to a 1:2000 mass-to-mass ratio of aFn14:PBNP. The mixture was contacted in the dark at RT for 3 h on an orbital shaker, after which 100 µL of 0.1 M hydroxylamine (Thermo Fisher Scientific, Waltham, MA, USA) was added to quench any remaining primary amine sites. aFn14-PBNP were again centrifuged at 22,000× *g* for 30 min at RT, resuspended in 1 mL DI H_2_O, and sonicated. This process was repeated twice for a total of three washes. The particles were then resuspended in the desired volume of sterile DI water and stored at 4 °C, protected from light.

### 2.3. Attachment Efficiency of aFn14 to PBNPs

The attachment efficiency of aFn14 to PBNPs was calculated based on the amount of aFn14 that remained unbound in the aFn14-PBNP synthesis supernatants. A standard curve of fluorescence intensity (λ_em_ = 490 nm, λ_ex_ = 525 nm) vs. known concentrations of FITC-conjugated aFn14 was generated using a SpectraMax i3x Multimode Microplate Reader (Molecular Devices, LLC, San Jose, CA, USA) (Figure S1). To determine the amount of aFn14 that did not bind to PBNPs after aFn14-PBNP synthesis, supernatants of the syntheses were collected and compared to the standard curve. The concentration and then mass of unbound aFn14 were calculated; the unbound mass was subtracted from the initial mass of aFn14 utilized for the synthesis to determine the final mass of aFn14 attached onto the PBNP collected. This value was then divided by the initial mass of aFn14 and multiplied by 100 to determine the attachment efficiency of the antibody.

### 2.4. Characterization of aFn14-PBNP

To quantify the size and charge of the aFn14-PBNPs, the hydrodynamic diameter and zeta potential of PNBPs and aFn14-PBNP were measured using dynamic light scattering (DLS) spectroscopy and zeta anemometry on a Zetasizer Nano ZS (Malvern Instruments, Malvern, UK). Optical characteristics of the constructs were measured via UV-Vis-NIR Spectroscopy using a Genesys 10S spectrophotometer and VISIONlite software (Thermo Fisher Scientific, Waltham, MA, USA). Attachment efficiency was measured via fluorescence spectroscopy as described in Section 2.3. To measure nanoparticle stability over time, DLS, zeta-anemometry, and fluorescence spectroscopy was performed at Day 0, +2, +4, +8, +16, and +20 following the initial particle synthesis. These physical characteristics were measured for every subsequent nanoparticle synthesis to assess whether the critical quality attributes of the nanoparticles were within acceptable standards for PBNP and aFn14-PBNP.

### 2.5. Cell Lines and Culture

Human U87 glioblastoma cells (ATCC, Manassas, VA, USA) were cultured in Eagle’s Minimal Essential Medium (EMEM; ATCC, Manassas, VA, USA) containing L-glutamine (Thermo Fisher Scientific, Waltham, MA, USA) supplemented with 10% fetal bovine serum (FBS; Thermo Fisher Scientific) and 1% penicillin/streptomycin antibiotic (Thermo Fisher Scientific, Waltham, MA, USA). Human U251 glioblastoma cells (NCI Developmental Therapeutics Program, Bethesda, MD, USA) were maintained in EMEM containing L-glutamine supplemented with 10% FBS, 1% non-essential amino acids (Thermo Fisher Scientific, Waltham, MA, USA), and 1% penicillin/streptomycin.

### 2.6. Characterization of the PTT Properties of aFn14-PBNP

In the clinic, PBNPs would be administered to the tumor site and then irradiated to ablate tumor tissue. In our in vitro protocol to mimic this procedure, samples treated with PBNPs are irradiated with an NIR laser at various laser powers, and the temperature and thermal dose are measured over time to characterize the impact of PBNP excitation and relaxation on the surrounding environment. This protocol is a simple and cost-effective way of modeling PTT in a laboratory setting under reproducible conditions.

In this study, 0.5 mL of water or 5 × 10^6^ U87 or U251 in 0.5 mL culture media were treated with 0.15 mg/mL PBNP or aFn14-PBNP. Note that 0.15 mg/mL aFn14-PBNP refers to the concentration of PBNP, not aFn14, to keep the total concentration of PBNPs in aFn14-PBNP consistent with the PBNP only condition. Suspensions were irradiated with an 808 nm NIR continuous wave collimated diode laser (Laserglow Technologies, Toronto, Canada) for 10 min. Temperature was measured using an infrared thermal camera (i7 thermal imaging camera, FLIR, Arlington, VA, USA) at 1 min intervals. The thermal dose administered was modulated by increasing the laser power administered in a stepwise manner. We utilized laser powers of 0.75 W, 1 W, 1.5 W, and 2 W, monitored using a power meter (Thorlabs, Newton, NJ, USA). The thermal dose output was then calculated using the CEM43°C formula, as shown in Equation (1):

(1) $$\mathrm{CEM}43{}^{\circ}C=\overset{n}{\underset{i=1}{\sum }}t_{i}*R^{\left(43-T_{i}\right)}$$

where *T_i_* is the *i*-th time interval, R is related to the temperature dependence of the rate of cell death (*R*(T < 43 °C) = 0.25, *R*(T > 43 °C) = 0.5) and T is the average temperature during time interval *T_i_* [45,46,47]. Cyclic heating/cooling studies were also performed on the nanoparticles for 3 cycles with the laser on and off for 10 min each for each cycle at a power of 2 W to elucidate the ability of the nanoparticles to withstand several cycles of laser illumination.

### 2.7. Elucidation of the Glioblastoma Tumor Cell Phenotype Post-PTT

PTT was administered to 0.5 mL suspensions containing 5 × 10^6^ U87 or U251 cells as described in Section 2.6. The treated cell suspension was then centrifuged, and the cells were suspended in their respective cell culture media and plated in 6 well plates at 37 °C for 24 h. After 24 h of undisturbed incubation, the cell culture media and the cells were harvested from the plates using TrypLE (Thermo Fisher Scientific, Waltham, MA, USA). Cells were then collected with media and transferred to 15 mL conical tubes and collected by centrifugation at 400× *g* for 5 min. The final cell pellets were then resuspended in PBS, and aliquoted accordingly for subsequent analysis. Viability was measured using a Luna cell counter (Logos Biosystems, Anyang, Korea) and acridine orange (Logos Biosystems, Anyang, Korea).

Intracellular levels of ATP were assessed to indirectly determine the amount of ATP released. Using the CellTiter-Glo Luminescent Viability Assay (Promega, Madison, WI, USA) and following the manufacturer’s protocol, 100 µL of cells at a concentration of 2.86 × 10^6^ mL in PBS from every condition were aliquoted into a 96-well opaque bottom plate. Once ATP assay reagents were at RT and mixed together, the ATP reagent was aliquoted at 100 µL per well to the cells. The plate was then covered in foil and placed on an orbital shaker for 2 min. The plate was then incubated at RT for 5–10 min before luminescence was measured via SpectraMax. PBS without cells was used as a control. All samples were performed in triplicate.

For flow cytometry analysis, 1 × 10^6^ cells were first stained with 1µL Zombie Violet^TM^ fixable viability dye, reconstituted at the manufacturer’s recommended concentration, for 20 min in 100 µL PBS (BioLegend, San Diego, CA, USA). After washing cells with flow buffer (PBS + 1% FBS), cells were resuspended in 100 µL flow buffer and blocked with 5 µL/tube Human TruStain FcX^TM^ (Fc Receptor Blocking solution, BioLegend, San Diego, CA, USA) for 10 min at 4 °C. The cells were then separated into three panels for staining, besides the fluorescence minus one (FMO), isotype, and unstained control groups: two cell surface staining panels and one cell surface and intracellular ICD staining panel. For the first panel, the following extracellular stains were added: GD2 (APC, clone 30-F11, 5 µL/tube), CD137L (PE, clone 5F4, 5 µL/tube), HLA-A,B,C (AlexaFluor700, clone W6/32, 5 µL/tube), B7-H3 (PE/Cy7, clone MIH42, 5 µL/tube), and PD-L1 (BV650, clone 29E.2A3, 5 µL/tube). For the second panel, the following extracellular stains were added: Fn14 (PE, clone ITEM-1, 2.5 µL/tube), CD86 (APC, clone BU63, 5 µL/tube), CD80 (BrilliantViolet650, clone 2D10, 5 µL/tube), CD40 (AlexaFluor700, clone 5C3, 5 µL/tube), and HLA-DR (PE/Cy7, clone L243, 5 µL/tube). All antibodies used in panels 1 and 2 were purchased from BioLegend. For the third panel, an antibody against calreticulin was added (PE, clone ab92516, 0.5 µL/tube) (Abcam, Cambridge, UK). All panels were stained for 30 min at 4 °C, fixed, and permeabilized using 250 µL/tube of 1× BD CytoFix/CytoPerm Solution (BD Biosciences, Franklin Lakes, NJ, USA) for 30 min at 4 °C. Panel 3 was then stained for intracellular HMGB1 (AlexaFluor647, clone ab195011, 2 µL/tube) (Abcam, Cambridge, UK) for 30 min at 4 °C. Flow cytometry was performed using the BD Biosciences Celesta Cell Analyzer or BD CytoFLEX (Indianapolis, IN, USA). Flow cytometry gating and analysis was performed using FlowJo software (v10.7.1, Ashland, OR, USA) and plotted using GraphPad Prism (v9.0.0, San Diego CA, USA).

### 2.8. Determining aFn14 Binding to U87 and U251 Cells via Flow Cytometry

U87 or U251 cells were stained with either the Fn14 antibody used for aFn14-PBNP synthesis (FITC, clone ITEM-4, Santa Cruz Biotechnology Inc., Dalles, TX, USA at manufacturer’s recommended concentration) or with the Fn14 antibody used for staining in the ICD panel (PE, clone ITEM-1, Biolegend, at manufacturer’s recommended concentration). Percent positive cells were gated on size and single cells.

### 2.9. Quantifying the Attachment of aFn14-PBNP to U87 Tumor Cells

Inductively Coupled Plasma Mass Spectroscopy (ICP-MS; Neptune Series High Resolution Multicollector ICP-MS; Thermo Fisher Scientific, Waltham, MA, USA) was performed in collaboration with the University of Maryland Plasma Mass Spectroscopy Lab. U87 GBM cells were grown in 6 well plates; aFn14-PBNP was then added to 1 × 10^6^ cells at a final concentration of 0.15 mg/mL for 15, 30, 45, 60, or 120 min. The cells were then rinsed with PBS, harvested using TrypLE, counted, washed, and all cells were resuspended in 1 mL PBS. The cells were then transferred to thick screw-top Teflon beakers and treated with high-purity concentrated nitric acid (OmniTrace Ultra, Supelco, Waltham, MA, USA) for 20 min at RT. The concentrated nitric acid was then diluted to a concentration of 2% (*v*/*v*) using DI water and digested further overnight at 60 °C. The samples were then filtered through a 40 µm cell strainer, transferred to a sealed tube and transported to the University of Maryland Plasma Mass Spectroscopy Lab. PBNP attachment was detected by probing for the ^57^Fe elemental isotope of non-radioactive iron, which is a component of the PBNPs.

### 2.10. Assessing the Efficacy of Using aFn14-PBNP for Targeted PTT of Glioblastoma Tumor Cells

We developed a protocol to determine whether aFn14-PBNPs provided an advantage over PBNPs in the context of a tumor where external forces, such as the circulatory or lymphatic system, may wash out unbound particles. 1 × 10^6^ U87 or U251 cells were incubated with 5.8 mg/mL PBNP, 5.8 mg/mL aFn14-PBNP, 3 µ/mL FITC-conjugated aFn14 (to match the concentration of aFn14 used in the aFn14-PBNP condition), or a vehicle control (water) for 2 h at 37 °C. Cells were then washed twice to remove unbound particles and/or antibody, resuspended in 500 µL media, and PTT-treated as described in Methods 2.6. Prior to commencing PTT, 50,000 cells from each condition were assessed for FITC expression via flow cytometry, as a measure of aFn14 attachment to the tumor cells. Cells were plated into 6 well plates (one well per sample, in 2 mL total cell culture media) and incubated at 37 °C. Viability was measured 24 h post-PTT using the Luna cell counter and acridine orange.

### 2.11. Statistical Analysis

Statistical significance for this study was determined using Welch’s *t*-test, and values with *p* < 0.05 were considered statistically significant. Descriptive statistics are reported as mean ± standard deviation. All statistical analyses were performed using GraphPad Prism.

## 3. Results

### 3.1. aFn14 Can Be Covalently Conjugated on PBNPs to Generate Stable aFn14-PBNPs

**Figure 1:**
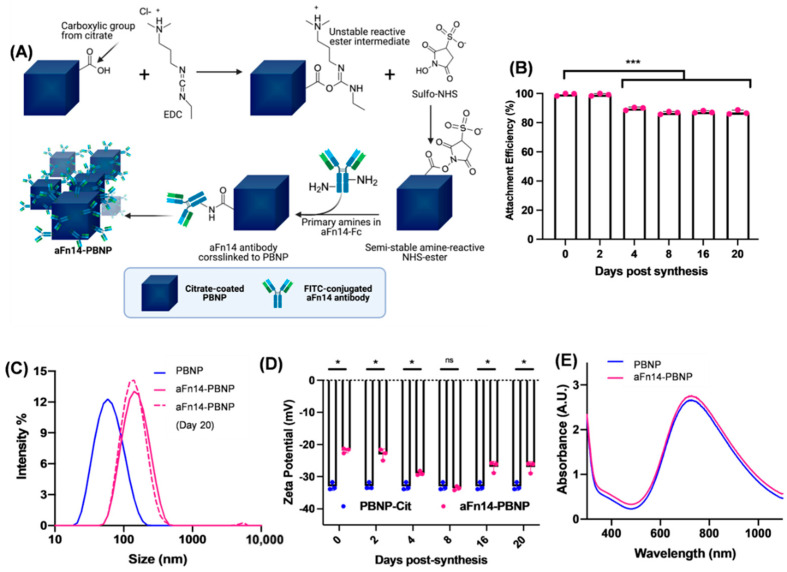
aFn14 can be covalently conjugated to PBNPs to generate stable aFn14-PBNPs. (**A**) Synthesis scheme used to generate aFn14-PBNPs, described in Methods 2.2. EDC-NHS chemistry was used to coat FITC-conjugated aFn14 antibody onto the surface of the PBNPs. The carboxyl groups from citrate on the surface of PBNPs are covalently conjugated to the amine groups on aFn14. (**B**) Attachment efficiency of aFn14 on to PBNPs over time. The initial attachment efficiency on Day 0 (99.4% SD 0.88%) did not significantly change on Day +2, but a statistically significant change was observed after Day +4 to a final attachment efficiency of 87.1% (SD 1.48%) by Day 20 (*p* < 0.0001). (**C**) Size distributions of PBNPs and aFn14-PBNPs as measured by dynamic light scattering. The size shift between PBNP and aFn14-PBNP indicates successful attachment of aFn14. There was no size change of aFn14-PBNP between Day 0 after synthesis (pink line) and Day 20 (dotted pink line), demonstrating long-term stability of aFn14-PBNPs. (**D**) Zeta potentials of PBNPs and aFn14-PBNPs. Statistically significant differences in the surface charge were observed between PBNPs and aFn14-PBNPs at all time points (except Day +8). This suggests the presence of the aFn14 antibody on the surface of aFn14-PBNPs. (**E**) UV-Vis-NIR spectra demonstrating the characteristic absorption peak of PBNP between 650–900 nm, which yields NIR responsiveness at 808 nm. Coating with aFn14 did not affect this absorption property. Welch’s *t*-test was utilized to test for statistical significance. * = *p* < 0.05 ** = *p* < 0.01 *** = *p* < 0.001; ns = not significant.

The size, charge, and UV-Vis-NIR absorption properties of PBNPs can affect their efficacy as PTT agents in the CNS. Nanoparticles that are too large (>200 nm) cannot effectively cross the blood–brain barrier [48,49] while nanoparticles with a positive surface charge can form nonspecific ionic adhesions to the negatively charged cell membranes and extracellular space of the CNS [50], resulting in unwanted off-target binding and/or toxicity. Additionally, a change in absorption properties of a PTT agent can alter the efficacy of photothermal energy conversion. Therefore, we aimed to synthesize nanoparticles that met the above design attributes. Using the scheme described in Section 2.2, the FITC-conjugated aFn14 antibody was covalently linked to the PBNPs to generate aFn14-PBNPs (Figure 1A). Based on the use of fluorescence spectroscopy to generate a standard curve for aFn14 quantification (Figure S1A,B), we calculated the attachment efficiency on the day of aFn14-PBNP synthesis (Day 0) to be 99.4% (SD 0.88%). Consequently, we estimated that the aFn14-PBNP nanoparticle had 0.497 µg of bound aFn14 per mg of PBNP. While the attachment efficiency of aFn14 on PBNP was initially very high, it decreased to 87.1% (SD 1.48%) by Day 20 post-synthesis, indicating that the conjugation resulted in the retention of the majority of aFn14 on the PBNPs for nearly three weeks. On Day 20, we estimated that aFn14-PBNP had 0.436 µg of bound aFn14 per mg of PBNP (Figure 1B).

Following synthesis, the hydrodynamic diameter of the aFn14-PBNPs increased from a mean of 58.7 nm for PBNPs to 122.4 nm for aFn14-PBNPs with polydispersity indices of 0.42 and 0.37, respectively (Figure 1C). Importantly, the mean hydrodynamic diameter and mean polydispersity index of the aFn14-PBNPs on Day 20 were unchanged compared to the freshly synthesized aFn14-PBNPs. Although the zeta potentials increased from −33.0 ± 1.2 mV for PBNPs alone to −23.1 ± 1.7 mV for aFn14-PBNPs (Figure 1D), indicative of the presence of the covalently attached antibody on the surface of the nanoparticles, the zeta potentials of aFn14-PBNP and PBNPs alone remained stable over 21 days. Pertinent to the PTT properties of PBNPs and aFn14-PBNPs, we measured their UV-Vis-NIR spectra (Figure 1E). Importantly, when matched in terms of PBNP concentrations, the UV-Vis-NIR spectrum of aFn14-PBNPs overlapped with that of PBNPs. Specifically, the aFn14-PBNP spectrum exhibited the characteristic absorption band of PBNP between 650–900 nm, indicating that conjugation of the antibody did not alter the absorbance spectrum attributed to the PBNPs in aFn14-PBNPs. These results indicate that our synthesis scheme yielded aFn14-PBNPs wherein aFn14 was successfully covalently attached onto the PBNPs with high attachment efficiency and that the resulting aFn14-PBNPs had stable size distributions, zeta potentials, and retained the absorption properties of PBNPs.

### 3.2. aFn14-PBNPs Retain the PTT Properties of PBNPs and Can Be Used to Administer a Range of Thermal Doses to U87 and U251 GBM Tumor Cells

**Figure 2:**
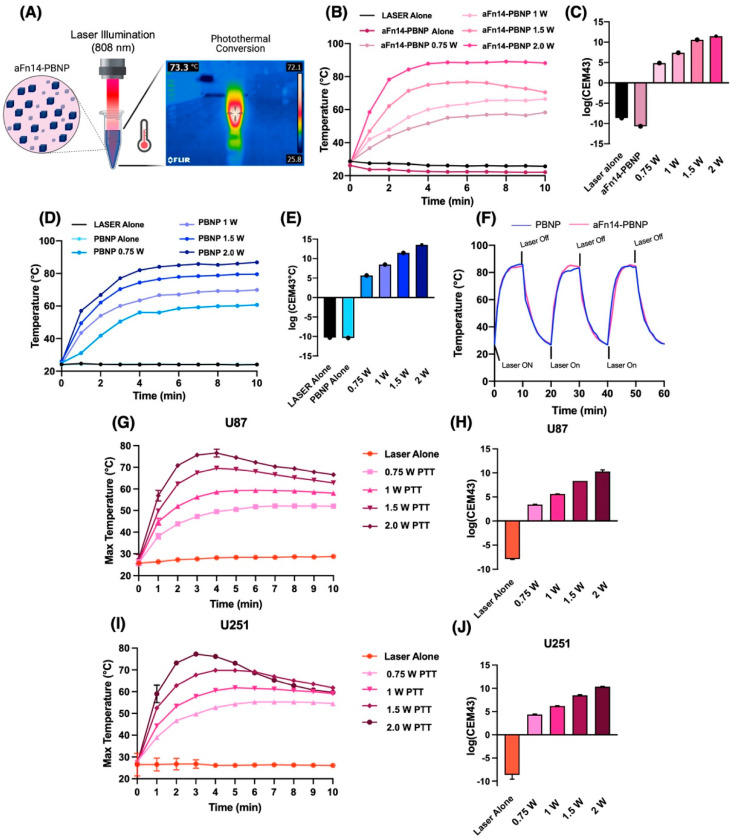
Conjugation to aFn14 does not alter the PTT properties of PBNPs, and aFn14-PBNPs can be used to administer a range of thermal doses to U87 and U251 GBM tumor cells. (**A**) Schematic of the PTT studies as described in Methods 2.6. Water or U87 or U251 cells in media were treated with 0.15 mg/mL PBNP or aF14-PBNP and then irradiated with an 808 nm laser. (**B**) Heating curves of aFn14-PBNP as a function of laser power. (**C**) Thermal doses delivered by aFn14-PBNPs as a function of laser power. (**D**,**E**) Heating curves (**D**) and thermal doses I delivered by PBNPs as a function of laser power. (**F**) Cyclic heating of PBNPs and aFn14-PBNPs over 3 heating-cooling cycles. (**B**–**F**) PTT properties of PBNP and aFn14-PBNP are similar, indicating that conjugation to aFn14 does not negatively affect PBNP PTT properties. (**G**) Heating curves (**H**) and thermal doses delivered to U87 GBM cells by aFn14-PBNPs as a function of laser power. (**I**) Heating curves (**J**) and thermal doses delivered to U251 GBM cells by aFn14-PBNPs as a function of laser power.

PBNPs absorb NIR radiation and reemit it as heat via phonon-to-phonon relaxations. This heating makes PBNPs useful as PTT agents to treat tumors, as the heat released can impart ablative thermal dosages onto surrounding tissues. For the aFn14-PBNPs to be functional as effective PTT agents, their photothermal properties must be retained after aFn14 conjugation. We therefore conducted studies assessing the PTT properties of both PBNPs and aFn14-PBNPs to determine whether conjugation to aFn14 alters these properties. Aqueous solutions of PBNPs or aFn14-PBNPs (both containing 0.15 mg/mL PBNPs) in DI water were irradiated with 808 nm NIR laser (Figure 2A) at various laser powers, and the temperatures of the water-nanoparticle systems were recorded every minute for 10 min. The aFn14-PBNP system exhibited laser power-dependent heating. Negligible heating was observed for the control system containing just water and irradiated with the laser at a 2 W (“LASER Alone”) and for the control system containing aFn14-PBNP but not irradiated (“aFn14-PBNP Alone”). The maximal temperatures attained for the aFn14-PBNP system were 88.2 °C for 2 W, 70.5 °C for 1.5 W, 66.4 °C for 1 W and 58.3 °C for 0.75 W laser power (Figure 2B). The corresponding thermal doses (log(CEM43)) delivered by the aFn14-PBNPs to the water following irradiation were 11.4 at 2 W, 10.6 at 1.5 W, 7.4 at 1.0 W, and 4.85 at 0.75 W (Figure 2C). The maximal temperatures attained for the PBNP system were 86.8 °C for 2 W, 79.6 °C for 1.5 W, 69.9 °C for 1 W, and 60.7 °C for 0.75 W laser power (Figure 2D). The corresponding thermal doses (log(CEM43)) delivered by the PBNPs to the water following irradiation were 13.65 at 2 W, 11.46 at 1.5 W, 8.46 at 1.0 W, and 5.69 at 0.75 W (Figure 2E). The heating curves and corresponding thermal doses generated with aFn14-PBNPs were comparable to those of PBNPs alone for all laser powers studied (Figure 2D,E). Cyclic heating and cooling studies, where the laser was turned on and off at specific intervals to allow heating and cooling, demonstrated comparable PTT characteristics between PBNP and aFn14-PBNP at equivalent PBNP concentrations as evidenced by the near overlap of their heating/cooling curves over three cycles (Figure 2F). These studies demonstrate that the addition of aFn14 did not alter the PTT properties of the aFn14-PBNPs, indicating the suitability of their use as PTT agents.

Next, we evaluated whether aFn14-PBNPs could heat tumor cells to temperatures consistent with those that are required for thermal ablation. PTT was conducted on 5 × 10^6^ U87 (Figure 2G,H) or U251 (Figure 2I,J) GBM tumor cell lines suspended in cell culture media containing 0.15 mg/mL PBNPs. Similar to the previous study, the heating curves and thermal dose delivered to each cell line by aFn14-PBNPs were assessed as a function of laser power. The maximal temperatures attained for U87 were 77 °C for 2 W, 69 °C for 1.5 W, 59 °C for 1 W and 52 °C for 0.75 W laser power (Figure 2G), with thermal doses of 9.4 at 2 W, 8.2 at 1.5 W, 6.5 at 1.0 W, and 4.9 at 0.75 W (Figure 2H). When U251 were treated with aFn14-PBNPs, similar temperatures and thermal doses were attained: 78 °C for 2 W, 68 °C for 1.5 W, 59 °C for 1 W and 51 °C for 0.75 W (Figure 2I), and thermal doses of 10.1 at 2 W, 8.1 at 1.5 W, 6.6 at 1.0 W, and 4.9 at 0.75 W (Figure 2J). The thermal doses attained during PTT with aFn14-PBNP were lower in the presence of GBM tumor cells for both cell lines as compared to those in water (e.g., 89 °C for aFn14-PBNP alone vs. 77 °C at 2 W for U87). This attenuation can be attributed to components present in the media (e.g., serum proteins) and is consistent with our observations when using uncoated PBNPs for PTT, as well as literature precedent [43,51,52,53]. However, the cells attained temperatures above those required to generate thermal ablation in treated tumor cells (>45 °C) at all laser powers tested for both GBM lines. Therefore, these results suggest that aFn14-PBNP can function as effective PTT agents for GBM tumor cells and can be used to administer a range of ablative thermal doses to these tumor cells.

### 3.3. PTT Using aFn14-PBNP Triggers Thermal and Immunogenic Cell Death in Treated GBM Tumor Lines

**Figure 3:**
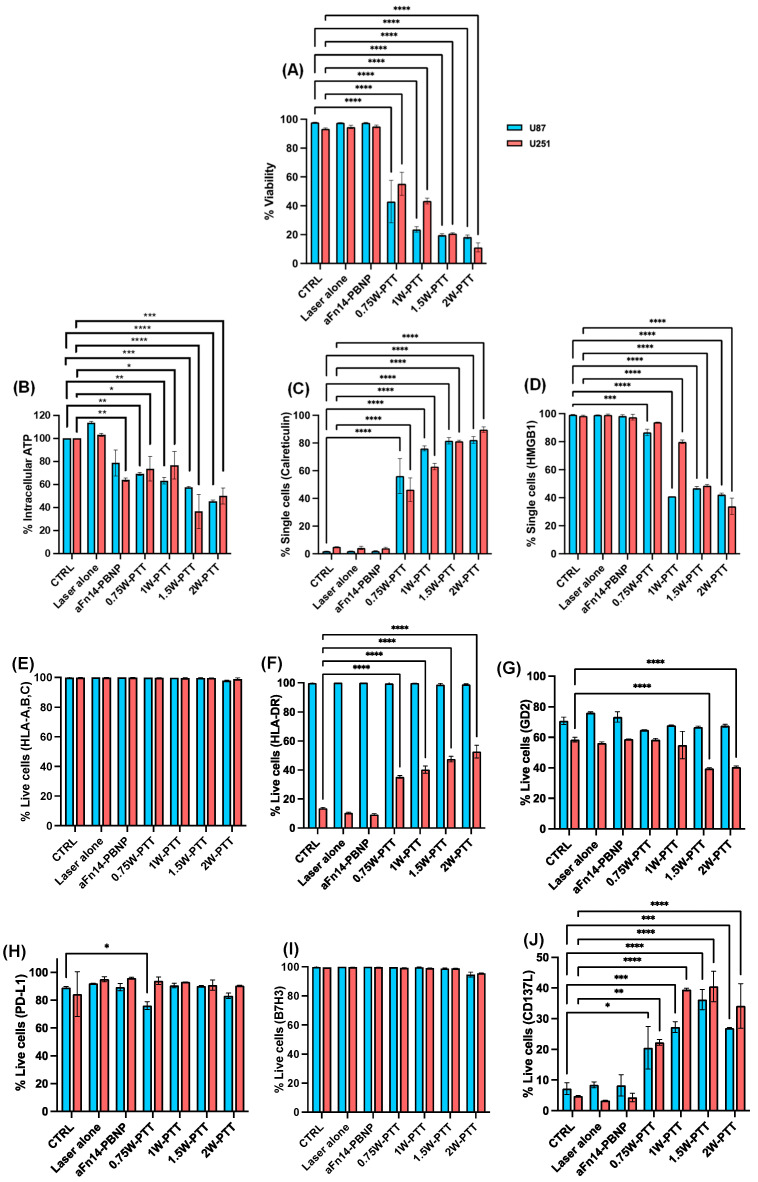

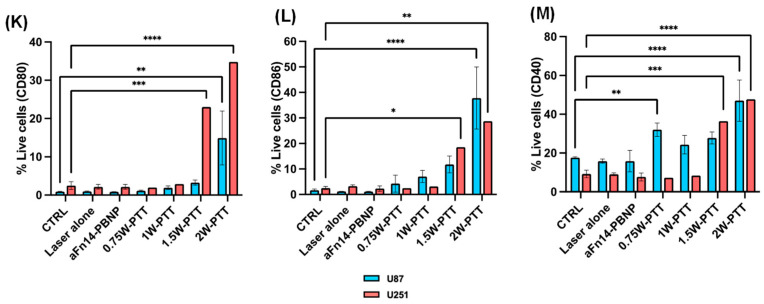
PTT using aFn14-PBNP triggers thermal and immunogenic cell death in treated GBM tumor lines. (**A**–**D**) U87 and U251 cells undergo thermal (**A**) and immunogenic cell death (**B**–**D**) after PTT with aFn14-PBNP, as noted by a decrease in cell viability (**A**), decrease in intracellular ATP (**B**), increase in surface calreticulin expression (**C**), and decrease in intracellular HMGB1 (**D**) with increasing laser powers (0.75–2 W). (**E**–**M**) PTT-induced changes in immunophenotype in GBM tumor lines, including MHC expression (**E**,**F**), tumor specific antigen expression (**G**), immune checkpoint inhibitor expression (**H**,**I**), and T cell costimulatory markers (**J**–**M**). CTRL = untreated cells not irradiated; laser alone = untreated cells irradiated with laser; aFn14-PBNP = aFn14-PBNP-treated cells not irradiated; remaining conditions are aFn14-PBNP-treated cells irradiated at indicated laser powers. * = *p* < 0.05; ** = *p* < 0.01; *** = *p* < 0.001; **** = *p* < 0.0001.

After undergoing PTT using aFn14-PBNPs as described in Figure 2G–J, the GBM tumor cells were plated for 24 h and then harvested for the analysis of PTT-triggered thermal and immunogenic cell death as well as immunophenotypic markers. PTT using aFn14-PBNP elicited thermally induced cell death in both GBM tumor lines as evidenced by a decrease in cellular viability in a laser power-dependent manner (Figure 3A). For U87 cells, the cellular viability decreased from 97.85% for aFn14-PBNP (without the laser) to 42.95% at 0.75 W, with the lowest viability of 18.45% observed at the highest laser power of 2 W. Similarly, for U251 cells, the cellular viability decreased from 95.00% for aFn14-PBNP (without the laser) to 55.25% at 0.75 W, with the lowest viability of 11.19% observed at the highest laser power of 2 W. For both tumor lines, the IC50 was attained at thermal doses of 4.72 for U87 and 4.86 for U251, respectively corresponding to a laser power of 0.75 W. These findings with aFn14-PBNP-based PTT in GBM tumor cells are consistent with our observations when using PBNPs for PTT in diverse tumor cell lines such as neuroblastoma, melanoma, and breast cancer [9,10,42,43]. We did not conduct studies assessing the effects of PBNPs alone (without the laser) as we have extensively observed that the PBNPs have a negligible effect on tumor cellular viability at the concentrations used for this study [9,10,42].

To assess the effect of PTT using aFn14-PBNP on eliciting ICD and immunophenotypic changes in treated GBM tumor cells, flow cytometry analysis of various surface and intracellular targets were conducted. The gating strategy for these analyses is elaborated in Figures S2–S5. PTT using aFn14-PBNPs induced ICD in both cell lines as measured by a decrease in total intracellular ATP levels (Figure 3B), the overexpression of calreticulin at the cell surface (Figure 3C, Figures S2A and S3A), and the release of HMGB1 from the cell as measured by a decrease in intracellular HGMB1 (Figure 3D, Figures S2B and S3B). The expression of all three biochemical correlates of ICD were most prominent for both U87 and U251 at laser powers of 1.5 W and higher.

PTT also induced changes in the tumor cell immunophenotype that may increase T cell immunity against these cells. MHC-I (HLA-A, B, and C) expression was high and retained in both U87 and U251 (Figure 3E, Figures S4A and S5A), and MHC-II expression (HLA-DR) remained unchanged in U87 cells but increased in U251 (Figure 3F, Figures S4B and S5B) as a function of laser power. Regarding expression of tumor-specific antigens, PTT caused a significant decrease in GD2 expression in U251 cells at 1.5 and 2.0 W but not in U87 cells, suggesting the retention of this tumor-specific antigen for this tumor line (Figure 3G, Figures S4E and S5E). In both cell lines, PTT maintained the expression of the immunosuppressive PD-L1 ligand (Figure 3H, Figures S4C and S5C) and no changes in expression of the immunosuppressive B7-H3 ligand were observed (Figure 3I, Figures S4D and S5D). Finally, PTT upregulated various T cell costimulatory markers in both cell lines, including CD137L (Figure 3J, Figures S4F and S5F), CD80 (particularly in U251; (Figure 3K, Figures S4G and S5G), CD86 (Figure 3L, Figures S4H and S5H), and CD40 (Figure 3M, Figures S4I and S5I), all in a dose-dependent manner. Overall, the changes induced by aFn14-PBNP were similar to those induced by PBNP, indicating that antibody coating did not drastically alter thermal or ICD-inducing properties of the PBNPs, except that expression of GD2 and CD137L remained higher in the aFn14-PBNP conditions compared to the PBNP-treated conditions (Figure S6). Together, these results demonstrate that PTT using aFn14-PBNPs generates thermal and immunogenic cell death, as well as favorably alters the surface immunophenotype of GBM tumor cells.

### 3.4. GBM Tumor Cells Differentially Express Fn14 That Can Be Successfully Targeted by aFn14-PBNPs

**Figure 4:**
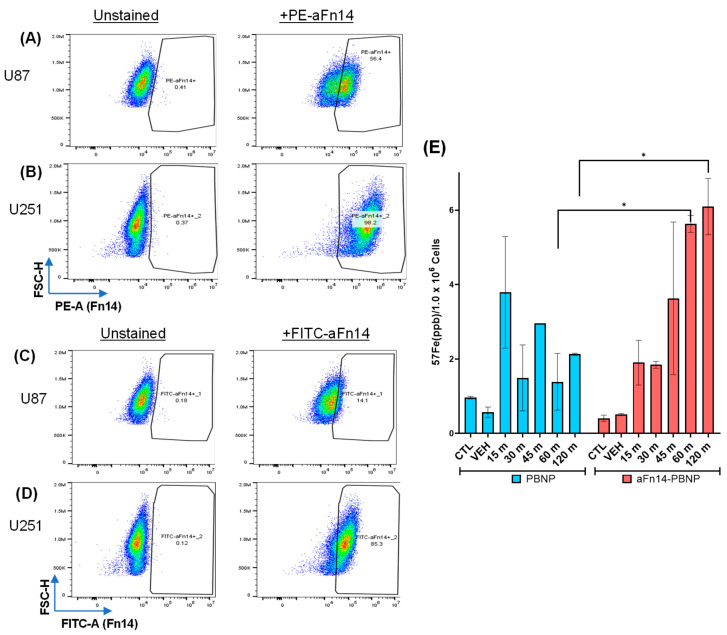
GBM tumor cells differentially express Fn14 that can be successfully targeted by aFn14-PBNPs. (**A**,**B**) Flow cytometry of (**A**) U87 and (**B**) U251 cells stained with PE-conjugated aFn14 antibody showing percent of cells expressing Fn14, compared to unstained. (**C**,**D**) Flow cytometry of both cell lines using the FITC-conjugated aFn-14 antibody compared to unstained. (**E**) ICP-MS of human U87 GBM cells incubated with 0.15 mg/mL PBNP (left) or aFn14-PBNP (right) for various time points, gating on the 57-Fe isotope. Each aFn14-PBNP group was compared to its respective PBNP group using a Welch’s *t*-test for significance. * = *p* < 0.05. CTL = control (U87 GBM cells alone). VEH = vehicle (U87 GBM cells with addition of water).

We conducted studies to assess the expression levels of Fn14 on the GBM tumor cells as described in Methods 2.8 (Figure 4). When probed with a PE-conjugated aFn14 antibody, both U87 and U251 showed expression of the Fn14 receptor via flow cytometry, with U87 expressing Fn14 on 56.4% of live cells (Figure 4A) and U251 expressing Fn14 on 98.2% of live cells (Figure 4B). However, when probed with the FITC-conjugated aFn14 antibody of a different clone, U87 showed expression of Fn14 only on 14.1% of live cells (Figure 4C) compared to U251 that demonstrated a higher expression of Fn14 on 85.3% of live cells (Figure 4D). Our results demonstrate that U251 consistently exhibit higher expression of Fn14 than U87 cells. The findings also demonstrate that although Fn14 is a suitable target for GBM, the antibody clone should be considered to facilitate success of tumor cell targeting with the nanoparticles. As our results will show later, however, even low aFn14 binding can retain enough PBNPs on cells to induce thermal death in response to PTT.

Next, we assessed whether aFn14-PBNPs can target U87 tumor cells using ICP-MS. In this study, we measured the amount of ^57^Fe elemental isotope in solution following acid digestion of U87 cells exposed to either aFn14-PBNP or PBNP for various contact times. Increased ^57^Fe, a main component of PBNPs, correlates with increased nanoparticle attachment to the surface of the GBM cells. U87 cells incubated with aFn14-PBNP nanoparticles showed an increase in signal over control or vehicle-treated cells. This increase was observed at 15 min (1.90 ± 0.60 ppb/1.0 × 10^6^ cells) and progressively increased with increasing contact time reaching a maximum at 120 min (6.10 ± 0.76 ppb/1.0 × 10^6^ cells), indicating stronger aFn14-PBNP binding to the U87 cells over time (Figure 4E). Importantly, there was a significant increase in ^57^Fe signal in the aFn14-PBNP condition after 60 and 120 min of incubation compared to U87 cells treated with PBNP alone (*p* = 0.0171 vs. *p* = 0.0177, respectively). Our results suggest that the addition of the aFn14 antibody increases the cellular targeting capacity of the PBNPs. The targeting was enhanced even in U87 GBM tumor cells that have lower Fn14 expression levels compared to U251, suggesting that the aFn14-PBNPs can be applied for targeted PTT against U87 cells. This experiment was not repeated in the U251 cells for cost saving and logistics purposes, but we expect similarly increased targeting of U251 cells by aFn14-PBNPs compared to PBNPs alone.

### 3.5. aFn14-PBNP Is an Effective Targeted PTT Agent for GBM Tumor Cells

**Figure 5:**
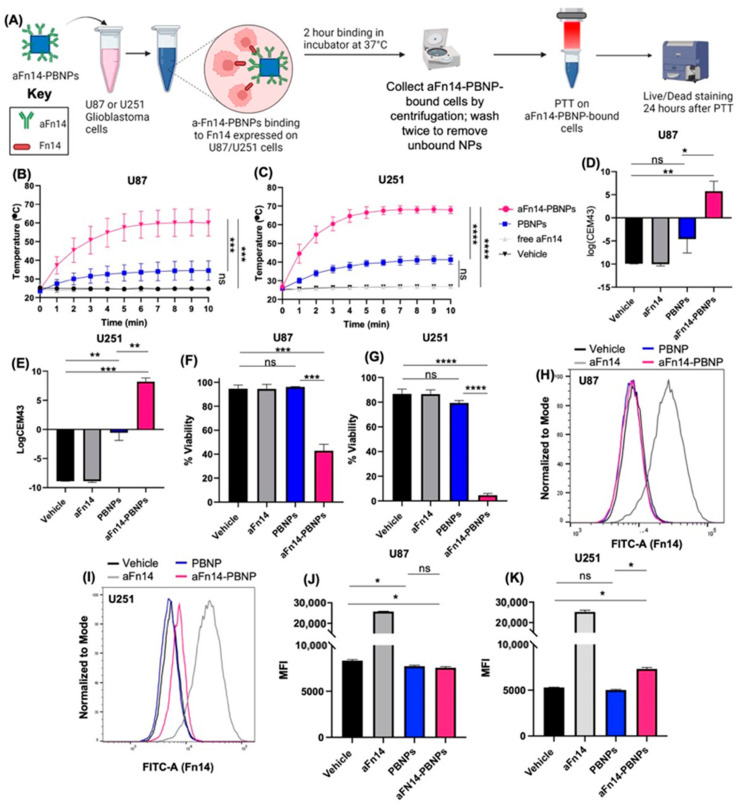
aFn14-PBNP is an effective targeted PTT agent for GBM tumor cells. (**A**) Experimental setup schematic, described in Methods 2.10: U87 or U251 cells were incubated with PBNPs, aFn14-PBNPs, free FITC-conjugated aFn14, or a vehicle control (water) for 2 h at 37 °C. Cells were then washed to remove unbound particles and irradiated with an 808 nm laser. Cells were plated for 24 h and analyzed via flow cytometry for viability. (**B**,**C**) Heating curves of U251 and U87 cell lines during PTT. (**D**,**E**) Thermal doses imparted on the cell lines during PTT. (**F**,**G**) Viability of tumor cells 24 h post-PTT. (**H**–**K**) Flow cytometric analysis done just before PTT of aFn14 binding in U87 and U251 cells. (**H**,**J**) No aFn14 binding is detectable in the aFn14-PBNP condition in U87 cells as indicated by the lack of a population shift or increase in MFI from the vehicle control to the aFn14-PBNP condition. (**I**,**K**) Some aFn14 is detectable in the aFn14-PBNP condition in U251 cells as indicated by a population shift and increase in MFI. (**B**–**K**) More aFn14-PBNPs remained bound to the U251 cells than to the U87 cells, which is reflected in the higher heating curves, thermal dose, and lower viability in this cell line (**B**–**G**,**I**,**K**) A one-way ANOVA was used to test for significance using Tukey’s HSD test for multiple comparisons. * = *p* < 0.05 ** = *p* < 0.01 *** = *p* < 0.001 **** = *p* < 0.0001. ns = not significant.

Thus far, we have demonstrated that conjugation to aFn14 does not negatively impact the photothermal properties of PBNPs, that aFn14-PBNPs can induce thermal and immunogenic cell death in two glioblastoma cell lines, and that aFn14-PBNPs can bind U87 cells. Next, we tested whether aFn14-PBNP-tumor cell binding provides a benefit over PBNPs when cells are washed to remove unbound particles before undergoing PTT (Figure 5). The goal of this study was to mimic, in a simple and cost-effective manner, how the blood or lymphatic system can wash PBNPs out of tumors.

In our ICP-MS study, we observed that U87 cells incubated with aFn14-PBNPs retained the largest number of particles 2 h after incubation (Figure 4E). Therefore, for this study, we incubated U87 and U251 cells with 5.8 mg/mL PBNPs, 5.8 mg/mL aFn14-PBNPs, 3 µg/mL free FITC-conjugated aFn14 (corresponding to the concentration of aFn14 used in the aFn14-PBNP condition), or a vehicle control (water) for 2 h at 37 °C. After this targeting step, the tumor cells were washed twice to remove any unbound nanoparticles and/or antibody. A sample of tumor cells were taken to measure FITC expression, which indicates aFn14-PBNP binding, just before PTT. The tumor cells were then irradiated with an 808 nm laser for 10 min and rested overnight (Figure 5A). Cell death was then assessed via flow cytometry. Because the purpose of the washing step was to remove untargeted particles after the contact time, the concentration of PBNPs and aFn14-PBNPs used had to be increased from that in Figure 2 and Figure 3 to attain a sufficient concentration of cell bound aFn14-PBNP concentration for thermal heating above 45 °C. Various concentrations and laser powers were tested to determine that 5.8 mg/mL was the optimal concentration for use in the U251 cell line for this experiment based on heating curves and thermal dose (Figure S7).

Both cell lines yielded higher heating curves (Figure 5B,C), thermal dose (Figure 5D,E), and cell death (Figure 5F,G) in response to PTT when incubated with aFn14-PBNPs compared to PBNPs alone, indicating that the antibody provided an advantage over PBNPs alone in effecting targeted PTT in the tumor cells. However, U251 cells consistently yielded higher heating curves (e.g., 68.25 °C for U251 vs. 60.20 °C for U87 at 2.0 W), thermal doses (e.g., 8.21 log(CEM43) for U251 vs. 5.73 log(CEM43) for U87 at 2.0 W), and cell death (42.90% viability for U251 vs. 4.63% viability for U87 at 2.0 W after 24 h) when treated with aFn14-PBNPs than U87 cells (Figure 5B–G). These results are consistent with previous findings that indicated that the antibodies used for targeting on aFn14-PBNP did not bind to the U87 cells as well as the U251 cells (Figure 4A–D). These results were also reflected in the flow cytometry analysis of the cells evaluated before PTT where only a negligible increase in the percent of FITC-positive U87 cells was observed after aFn14-PBNP treatment compared to PBNP treatment (Figure 5H,J). In contrast, there was a much more noticeable increase in the percent of FITC-positive U251 cells treated with aFn14-PBNP compared to PBNP (Figure 5I,K). However, despite the negligible presence of aFn14-PBNP on U87 as measured via flow cytometry, the U87 cells treated with aFn14-PBNPs still yielded a higher thermal dose (5.74 log(CEM43) at 2.0 W) and significant decrease in viability (42.90% live) compared to those treated with PBNP (−4.59 log(CEM43) at 2.0 W and 96.05% live) post-PTT. These results indicate that even an amount of antibody binding that is too low to be detected by flow cytometry can generate heating and elicit thermally induced cell death. These trends were further accentuated in studies with U251 tumor cells where treatment with aFn14-PBNPs yielded an even higher thermal dose (8.21 log(CEM43) at 2.0 W) compared to the PBNP-treated U251 cells (−0.60 log(CEM43) at 2.0 W) and an even larger significant decrease in U251 viability following PTT (79.4% for PTT with PBNP vs. 4.63% for PTT with aFn14-PBNP). Together, these results clearly indicate that aFn14-PBNPs are capable of inducing thermal death in response to PTT as a function of Fn14-based targeting. Overall, because they are retained on the surface of aFn14-expressing cells, the aFn14-PBNPs provide an advantage over PBNPs alone in an environment where the particles are at risk of being washed out of the tumor by external forces.

## 4. Discussion

For the past two decades, PTT has been widely investigated as an experimental ablative cancer therapy. While PTT for GBM in vitro and in vivo has been reported, including using gold nanorods [54], graphene oxide [55], and indocyanine green nanoparticles [56], to the best of our knowledge, this is the first study that utilizes a PBNP-based platform for treating GBM tumor cells. Additionally, we also report the first successful synthesis of a PBNP therapeutic modality targeting Fn14, a cytokine receptor highly upregulated in GBM compared to surrounding tissue in the tumor microenvironment [23,24,30,57,58]. Not only is our method novel, it is also simple and cost-effective. Required materials consist exclusively of inexpensive reagents commonly found in a cell culture and nanomaterials laboratory: FeCl_3_•6H_2_O, citric acid, K_4_[Fe(CN)_6_]•3H_2_O, MES buffer, EDC solution, sulfo-NHS solution, and the aFn14 antibody. The synthesis and conjugation protocols are straightforward and take just one day to complete. From a practical standpoint, these qualities make our method an attractive one to pursue further as it can be widely replicated and adapted for little cost (detailed protocols are provided in the Supplementary Methods).

Our facile and cost-effective synthesis scheme yielded aFn14-PBNPs that retained the targeting antibody (aFn14), exhibited stable size distributions and surface charges for up to 21 days, and maintained the optical properties (UV-Vis-NIR spectrum) of unconjugated PBNPs (Figure 1). aFn14-PBNPs also retained the photothermal properties of unconjugated PBNPs, exhibiting similar laser power-dependent heating and the ability to administer a range of thermal doses to the GBM tumor cell lines U87 and U251 in vitro (Figure 2). These effects are due to the photothermal mechanism of laser excitation of the particles followed by phonon-phonon relaxations that release heat into the surroundings. The synthesis of nanoparticles with consistent critical quality attributes (e.g., size distributions, stability, PTT properties) are imperative for use in preclinical and clinical studies.

PTT using aFn14-PBNP elicited thermally induced and immunogenic cell death in both U87 and U251 tumor cells, with the biochemical correlates of ICD being expressed at higher levels after irradiation with higher laser powers (1.5 W and 2 W) (Figure 3). Further, PTT with aFn14-PBNP resulted in a unique tumor cellular immunophenotype consisting of thermal dose-dependent enhanced expression of CD137L, CD80, CD86, and CD40. These cellular and molecular signatures are indicative of a tumor cell death phenotype that can be combined with complementary immunotherapies to generate a robust antitumor immune response and potentiate the abscopal effect. To this end, aFn14-PBNP-PTT should be investigated in conjunction with co-localized or co-administered immunotherapeutic agents including immunological adjuvants (e.g., toll-like receptor agonists), monoclonal antibodies (e.g., anti-PD-1), and/or immune cell therapies for treating GBM in syngeneic animal models of the disease. However, it is important to note that the enhanced expression of these immune markers on the GBM cells would need to be verified in in vivo GBM tumors after PTT to assess the therapeutic potential of these PTT-induced molecular modulations. The success of previous combination approaches, including our own [9,40,43,59], in syngeneic models of neuroblastoma, melanoma, and breast cancer provide us with the compelling rationale to pursue these studies in the context of GBM.

Conjugation to aFn14 also provided a benefit to PBNPs by retaining PBNPs on the surface of aFn14-expressing cells, as evidenced by flow cytometry, ICP-MS, and PTT studies (Figure 4 and Figure 5). We observed that aFn14-PBNPs were more effective in targeting U251 cells compared to U87 cells, which was likely due to the decreased aFn14 binding in the U87 compared to U251 (Figure 4C,D). Despite this lower binding, both ICP-MS and PTT conducted after washing unbound particles from cells demonstrated that the aFn14-PBNPs bound more strongly to U87 than unconjugated PBNPs (Figure 4 and Figure 5), resulting in approximately 12 times more killing when PTT was conducted on the U87 post-wash (5% vs. 60% killing) (Figure 5F). These studies provide evidence for the benefit of antibody-mediated targeting.

However, our studies are not without limitations. First, it is possible that aFn14 binding to Fn14 could induce TWEAK-like signaling along the NFkB pathway, leading to a more metastatic tumor phenotype. These results occurred in the aFn14 gold nanoparticle study conducted by Aido et. al. [29]. In this situation, PTT may mitigate much of the potential for metastatic conversion, as most of the cells would be ablated soon after contact. However, the exact binding location of the antibody on aFn14 would need to be considered and validated in order to confirm that a blocking, not activating, effect is achieved by antibody-Fn14 binding. Second, to ensure the success of our PBNP-based approach for tumor cells with similar or lower Fn14 expression levels as U87, we will explore other available antibody clones or targeting moieties, such as aptamers, as targeting agents on our nanoparticles [60,61,62]. We also cannot discount the fact that lower Fn14-expressing tumor cells (i.e., U87 in this study) may require a higher loading of the targeting antibody to ensure efficient binding. However, synthesis with increased antibody loading will have to be performed in the context of maintaining the critical quality attributes of the final nanoparticles in terms of size, stability, and other functional properties. Third, our in vitro binding studies (Figure 4 and Figure 5) are not an exact replication of the forces occurring within the tumor. An in vivo xenograft model would provide a more accurate representation of the kinetics of how PBNPs may be washed out of tumors.

If in vivo models are to be used in the future to continue these studies, we will need to consider how the aFn14-PBNPs will be administered, such as intratumorally or intravenously. If administered intravenously, we will need to monitor where the particles accumulate in organs besides the tumor, such as in the liver or kidney. We will also need to test our theory that the particles will have the proper size distribution to cross the blood–brain barrier. For either administration route, we will also need to take into consideration tumor volume, as the accumulation of the aFn14-PBNPs at the tumor site can be highly variable depending on this parameter. Finally, to administer this targeted approach for animal models of GBM, the nanoparticles and the laser will have to be implemented using technologies and methods common to neurosurgery. One option to administer the nanoparticles to the CNS is to use convection enhanced delivery to place the aFn14-PBNP in the vicinity of the GBM tumors in the CNS [63,64]. This will avoid having to utilize systemic infusion of the nanoparticles that will then have to cross the blood–brain barrier. The second component is to excite the delivered nanoparticles with an interstitially placed laser fiber similar to those clinically used for laser interstitial thermal therapy [65,66,67,68]. Our research group has completed a thorough evaluation of this interstitial PTT approach (we term this as I-PTT) in subcutaneous models of neuroblastoma and proposes to implement I-PTT for GBM in animal models in the near future.

## 5. Conclusions

In conclusion, we demonstrate that aFn14 can be conjugated to PBNPs to generate aFn14-PBNPs with quality attributes (size, charge, stability, UV-Vis-NIR) that retain PBNP photothermal properties. We also demonstrate that aFn14-PBNP-PTT elicits thermal and immunogenic cell death in GBM tumor cells. We show that aFn14-PBNP can target GBM tumor cells based on Fn14 targeting capabilities of the PBNPs. Finally, we show that when unbound particles are removed from cells pre-PTT, aFn14-PBNP-PTT is more effective than PBNP-PTT in triggering GBM tumor cell death. Altogether, these findings present a novel and cost-effective aFn14-PBNP nanotherapeutic for inducing thermal and immunogenic cell death in GBM tumor cells. Future studies might consider using an in vivo model, combination therapies, or interstitially administered PTT.

## Supplementary Materials

The following are available online at https://www.mdpi.com/article/10.3390/nano12152645/s1, Section S1: Supplementary Figures; Figure S1: Attachment efficiency of aFn14 to PBNPs based on (a) standard curve of fluorescence intensity compared to (b) fluorescence intensity of synthesis supernatants; Figure S2: Expression levels of calreticulin and HMGB1 in U87 tumor cells. (a) Gating strategy. (b,c) Flow cytometry histogram and dot plots of cell surface expression of (b) calreticulin (c) and intracellular expression of HMGB1 in the human U87 GBM cell line following PTT with aFn14-PBNP at various laser powers; Figure S3: Expression levels of calreticulin and HMGB1 in U251 tumor cells; Figure S4: Flow cytometry histograms and dot plots of expression of (a) HLA-A,B,C, (b) HLA-DR, (c) PD-L1, (d) B7H6, (e) GD2, (f) CD137L, (g) CD80, (h) CD86, and (i) CD40 in the human U87 GBM cell line both before and following PTT with aFn14-PBNP at various laser powers; Figure S5: Flow cytometry histograms and dot plots of expression of the above markers in the human U251 GBM cell line; Figure S6. PTT using aFn14-PBNP triggers thermal and immunogenic cell death in a manner similar to PBNPs in U87 tumor cells, (a) viability, (b,c) MHC expression, (d) tumor specific antigen expression (e,f) immune checkpoint inhibitor expression, (g–j) T cell costimulatory markers; Figure S7: Determination of the concentration of aFn14-PBNP needed for the targeted PTT assay. (a) Schematic. (b,c) Heating curves (b) and the corresponding thermal doses administered (c) to U251 after incubation with various concentrations of aFn14-PBNPs during PTT. (d,e) Heating curves (d) and corresponding thermal doses administered (e) to U251 after incubation with various concentrations of PBNPs during PTT. Section S2: Step-by-Step Protocols; 2.1. Synthesis of PBNPs; 2.2. Synthesis of bioconjugated anti-Fn14-PBNP; 2.3. Attachment efficiency of anti-Fn14 to PBNPs; 2.4. Characterization of anti-Fn14-PBNP; 2.5. Cell lines and culture; 2.6. Characterization of the PTT properties of anti-Fn14-PBNP; 2.7. Elucidation of the glioblastoma tumor cell phenotype post-PTT; 2.8. Determining anti-Fn14 binding to U87 and U251 cells via flow cytometry; 2.9. Quantifying the attachment of anti-Fn14-PBNP to U87 tumor cells; 2.10. Assessing the efficacy of using anti-Fn14-PBNP for targeted PTT of glioblastoma tumor cells.
