# Supplementary material for: Anti-Fn14-Conjugated Prussian Blue Nanoparticles as a Targeted Photothermal Therapy Agent for Glioblastoma
Source: Nanomaterials (Basel). 2022 Aug 1;12(15):2645. doi: 10.3390/nano12152645 (PMC9370342; doi:10.3390/nano12152645)
Supplement: Supplementary file 1 [file nanomaterials-12-02645-s001.zip › nanomaterials-1798645-supplementary.pdf]

nanomaterials

MDPI

Supplementary Material:

Anti-Fn14-Conjugated Prussian Blue Nanoparticles as a Targeted Photothermal Therapy Agent for Glioblastoma

Nicole F. Bonan [1,2,†], Debbie K. Ledezma [1,2,†], Matthew A. Tovar [1,3,†], Preethi B. Balakrishnan [1,†], Rohan Fernandes [1,2,3,4,*]

[1] George Washington Cancer Center, George Washington University, Washington, DC, USA
[2] Institute for Biomedical Sciences, George Washington University, Washington, DC, USA
[3] School of Medicine and Health Sciences, George Washington University, Washington, DC, USA
[4] Department of Medicine, George Washington University, Washington, DC, USA
* Correspondence: rfernandes@gwu.edu
† These authors contributed equally to this work.

This Supplementary Materials document contains:

Section S1: Supplementary Figures
Figure S1: Attachment efficiency of aFn14 to PBNPs.
Figure S2: Expression levels of calreticulin and HMGB1 in U87 tumor cells.
Figure S3: Expression levels of calreticulin and HMGB1 in U251 tumor cells.
Figure S4: Flow cytometry histograms and dot plots of expression of: HLA-A,B,C, HLA-DR, PD-L1, B7H6, GD2, CD137L, CD80, CD86, and CD40 in the human U87 GBM cell line both before (CTRL) and following PTT with aFn14-PBNP at various laser powers.
Figure S5: Flow cytometry histograms and dot plots of expression of: HLA-A,B,C, HLA-DR, PD-L1, B7H6, GD2, CD137L, CD80, CD86, and CD40 in the human U251 GBM cell line both before (CTRL) and following PTT with aFn14-PBNP at various laser powers.
Figure S6. PTT using aFn14-PBNP triggers thermal and immunogenic cell death in a manner similar to PBNPs in U87 tumor cells.
Figure S7: Determination of the concentration of aFn14-PBNP needed for the targeted PTT assay.

Section S2: Step-by-Step Protocols
S**2.1.** Synthesis of PBNPs
S**2.2.** Synthesis of bioconjugated anti-Fn14-PBNP
S**2.3.** Attachment efficiency of anti-Fn14 to PBNPs
S**2.4.** Characterization of anti-Fn14-PBNP
S**2.5.** Cell lines and culture
S**2.6.** Characterization of the PTT properties of anti-Fn14-PBNP
S**2.7.** Elucidation of the glioblastoma tumor cell phenotype post-PTT
S**2.8.** Determining anti-Fn14 binding to U87 and U251 cells via flow cytometry
S**2.9.** Quantifying the attachment of anti-Fn14-PBNP to U87 tumor cells
S**2.10.** Assessing the efficacy of using anti-Fn14-PBNP for targeted PTT of glioblastoma tumor cells

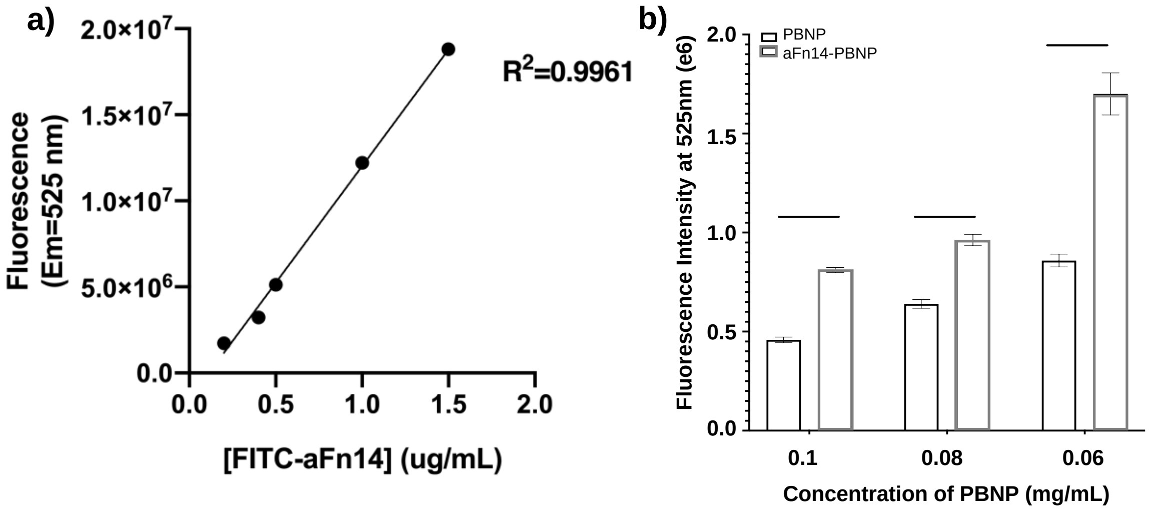

Figure S1: Attachment efficiency of aFn14 to PBNPs. The attachment efficiency was calculated based on the concentration of unbound antibody in the synthesis supernatants. (a) A standard curve of fluorescence intensity (λ_{em}= 490 nm, λ_{ex}= 525 nm) as a function of FITC-conjugated aFn14 concentration. (b) Fluorescence intensity (λ_{em}= 490 nm, λ_{ex}= 525 nm) of aFn14-PBNP synthesis supernatants at various concentrations of PBNPs. Fluorescence intensity of PBNPs alone is also shown as a negative control. The fluorescence intensity of the supernatants were compared to the standard curve in order to determine the attachment efficiency of aFn14 to PBNPs.

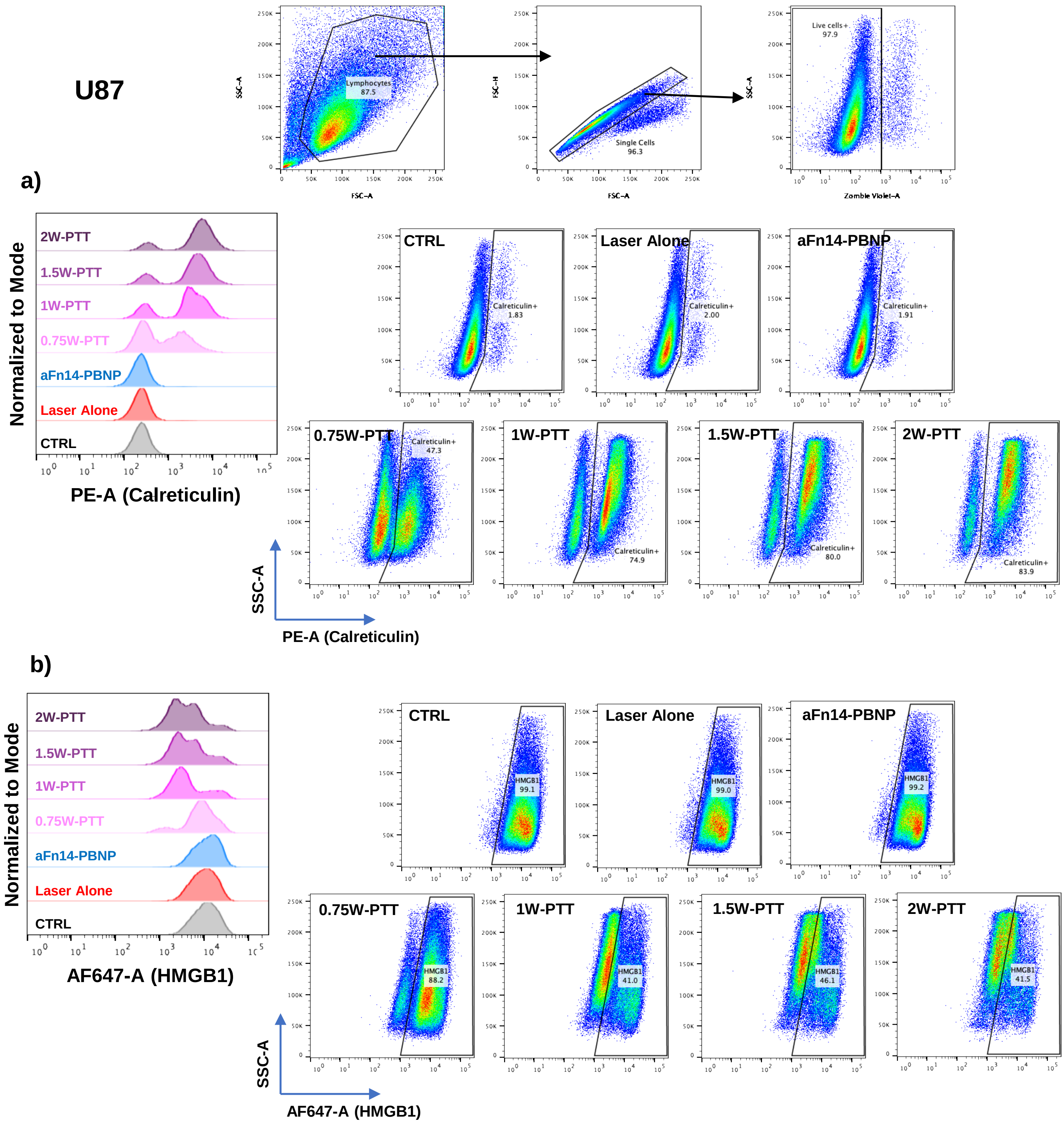

Figure S2: Expression levels of calreticulin and HMGB1 in U87 tumor cells. (a-b) Flow cytometry histogram and dot plots of cell surface expression of calreticulin (a) and intracellular expression of HMGB1 (b) in the human U87 GBM cell line following PTT with aFn14-PBNP at various laser powers. FMO = fluorescence minus one; CTRL = untreated cells; Laser alone = untreated cells irradiated with laser; aFn14-PBNP = cells treated with aFn14-PBNP but not irradiated; remaining conditions were treated with aFn14-PBNP and then irradiated at the indicated laser powers.

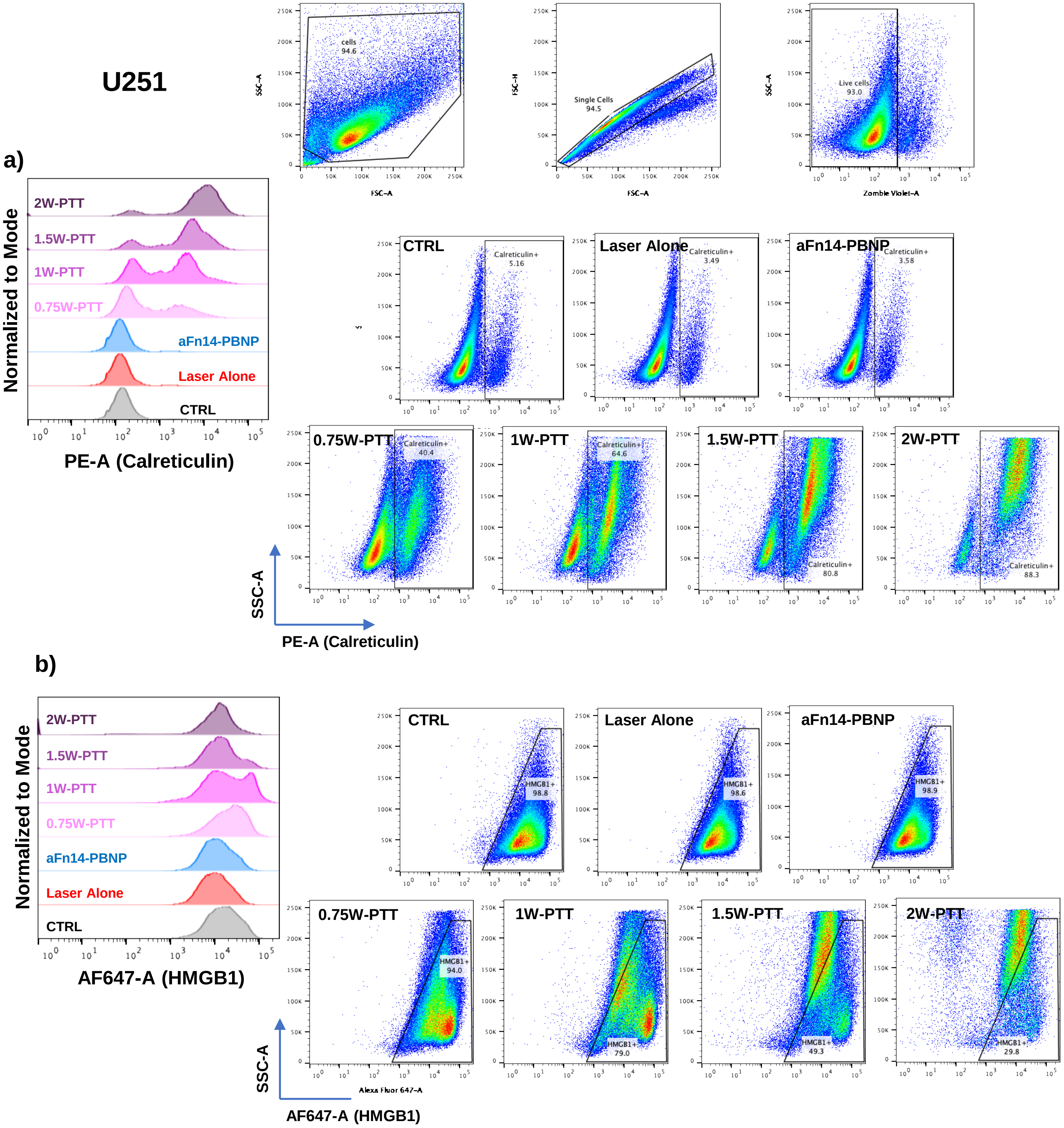

Figure S3: **Expression levels of calreticulin and HMGB1 in U251 tumor cells.** (a-b) Flow cytometry histogram and dot plots of cell surface expression of calreticulin (a) and intracellular expression of HMGB1 (b) in the human U251 GBM cell line following PTT with aFn14-PBNP at various laser powers. FMO = fluorescence minus one; CTRL = untreated cells; Laser alone = untreated cells irradiated with laser; aFn14-PBNP = cells treated with aFn14-PBNP but not irradiated; remaining conditions were treated with aFn14-PBNP and then irradiated at the indicated laser powers.

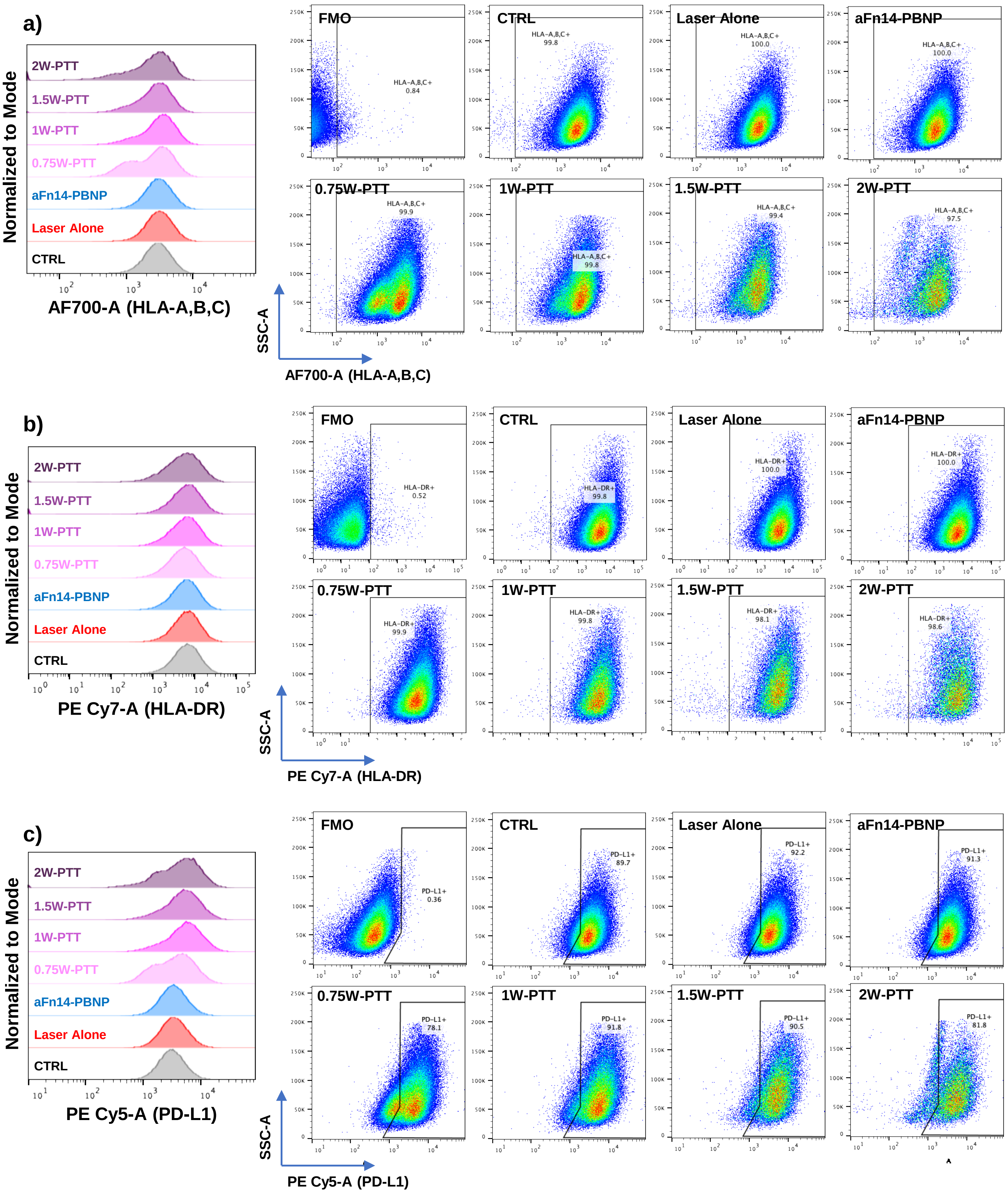

a)
2W-PTT
1.5W-PTT
1W-PTT
0.75W-PTT
aFn14-PBNP
Laser Alone
CTRL
Normalized to Mode
AF700-A (HLA-A,B,C)
FMO
HLA-A,B,C+ 0.84
CTRL
HLA-A,B,C+ 99.8
Laser Alone
HLA-A,B,C+ 100.0
aFn14-PBNP
HLA-A,B,C+ 100.0
0.75W-PTT
HLA-A,B,C+ 99.9
1W-PTT
HLA-A,B,C+ 99.8
1.5W-PTT
HLA-A,B,C+ 99.4
2W-PTT
HLA-A,B,C+ 97.5
SSC-A
AF700-A (HLA-A,B,C)
b)
2W-PTT
1.5W-PTT
1W-PTT
0.75W-PTT
aFn14-PBNP
Laser Alone
CTRL
Normalized to Mode
PE Cy7-A (HLA-DR)
FMO
HLA-DR+ 0.52
CTRL
HLA-DR+ 99.8
Laser Alone
HLA-DR+ 100.0
aFn14-PBNP
HLA-DR+ 100.0
0.75W-PTT
HLA-DR+ 99.9
1W-PTT
HLA-DR+ 99.8
1.5W-PTT
HLA-DR+ 98.1
2W-PTT
HLA-DR+ 98.6
SSC-A
PE Cy7-A (HLA-DR)
c)
2W-PTT
1.5W-PTT
1W-PTT
0.75W-PTT
aFn14-PBNP
Laser Alone
CTRL
Normalized to Mode
PE Cy5-A (PD-L1)
FMO
PD-L1+ 0.36
CTRL
PD-L1+ 89.7
Laser Alone
PD-L1+ 92.2
aFn14-PBNP
PD-L1+ 91.3
0.75W-PTT
PD-L1+ 78.1
1W-PTT
PD-L1+ 91.8
1.5W-PTT
PD-L1+ 90.5
2W-PTT
PD-L1+ 81.8
SSC-A
PE Cy5-A (PD-L1)

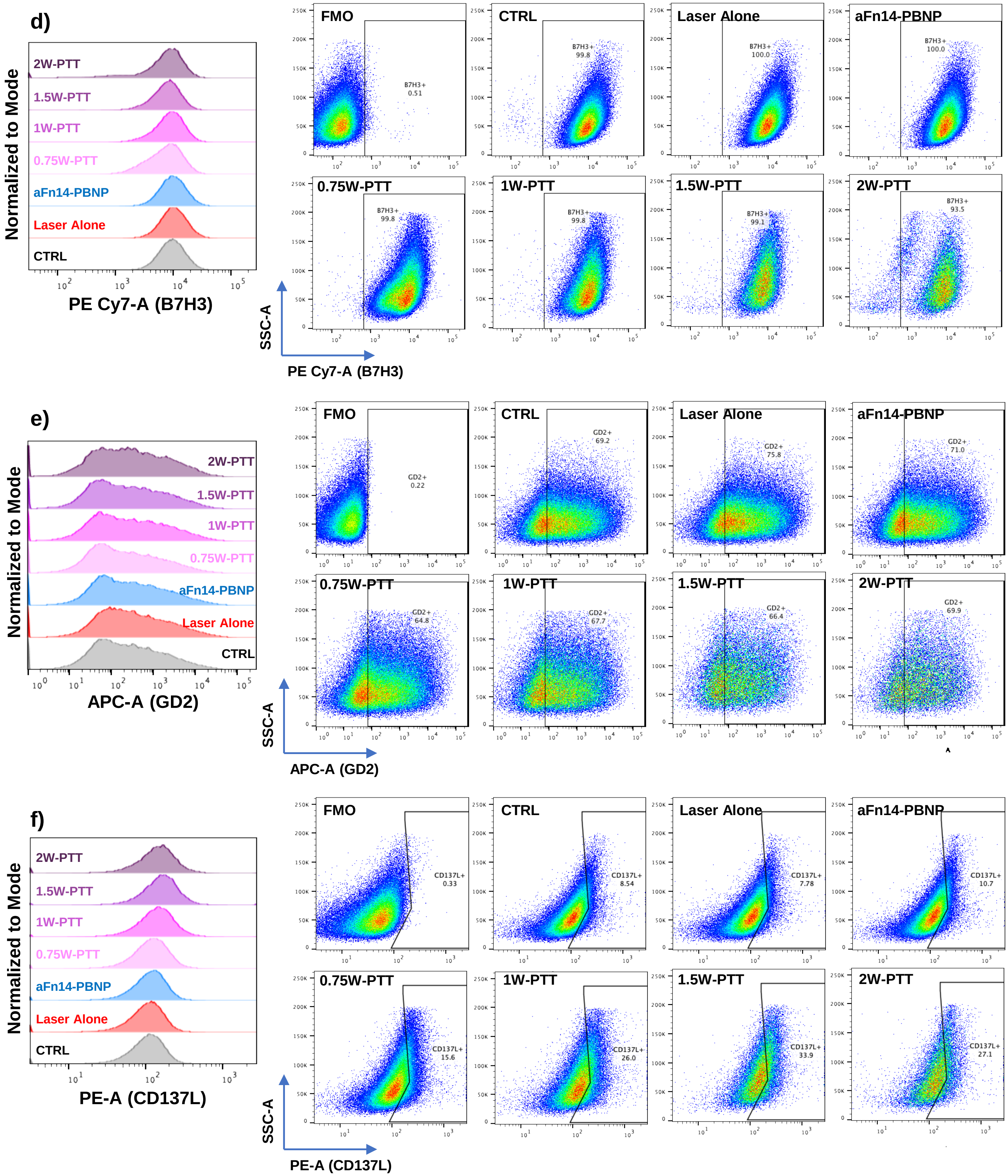

d)
2W-PTT
1.5W-PTT
1W-PTT
0.75W-PTT
aFn14-PBNP
Laser Alone
CTRL
Normalized to Mode
PE Cy7-A (B7H3)
FMO
B7H3+ 0.51
CTRL
B7H3+ 99.8
Laser Alone
B7H3+ 100.0
aFn14-PBNP
B7H3+ 100.0
0.75W-PTT
B7H3+ 99.8
1W-PTT
B7H3+ 99.8
1.5W-PTT
B7H3+ 99.1
2W-PTT
B7H3+ 93.5
SSC-A
PE Cy7-A (B7H3)
e)
2W-PTT
1.5W-PTT
1W-PTT
0.75W-PTT
aFn14-PBNP
Laser Alone
CTRL
Normalized to Mode
APC-A (GD2)
FMO
GD2+ 0.22
CTRL
GD2+ 69.2
Laser Alone
GD2+ 75.8
aFn14-PBNP
GD2+ 71.0
0.75W-PTT
GD2+ 64.8
1W-PTT
GD2+ 67.7
1.5W-PTT
GD2+ 66.4
2W-PTT
GD2+ 69.9
SSC-A
APC-A (GD2)
f)
2W-PTT
1.5W-PTT
1W-PTT
0.75W-PTT
aFn14-PBNP
Laser Alone
CTRL
Normalized to Mode
PE-A (CD137L)
FMO
CD137L+ 0.33
CTRL
CD137L+ 8.54
Laser Alone
CD137L+ 7.78
aFn14-PBNP
CD137L+ 10.7
0.75W-PTT
CD137L+ 15.6
1W-PTT
CD137L+ 26.0
1.5W-PTT
CD137L+ 33.9
2W-PTT
CD137L+ 27.1
SSC-A
PE-A (CD137L)

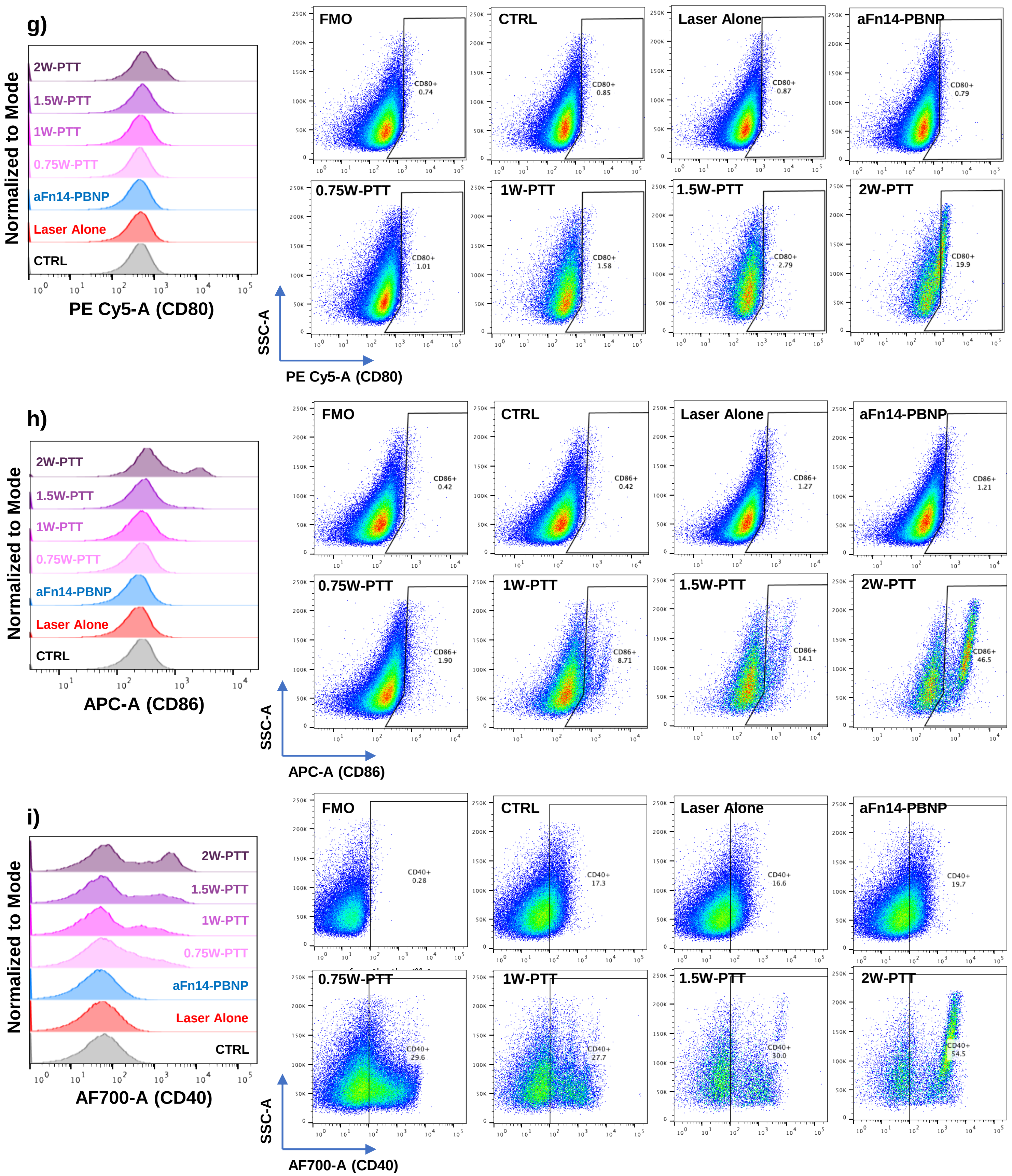

Figure S4: **Flow cytometry histograms and dot plots of expression** of (a) HLA-A,B,C, (b) HLA-DR, (c) PD-L1, (d) B7H6, (e) GD2, (f) CD137L, (g) CD80, (h) CD86, and (i) CD40 in the human U87 GBM cell line both before (CTRL) and following PTT with aFn14-PBNP at various laser powers. FMO = fluorescence minus one; CTRL = untreated cells; Laser alone = untreated cells irradiated with laser; aFn14-PBNP = cells treated with aFn14-PBNP but not irradiated; remaining conditions were treated with aFn14-PBNP and then irradiated at indicated laser power. See Figure S2A for gating strategy.

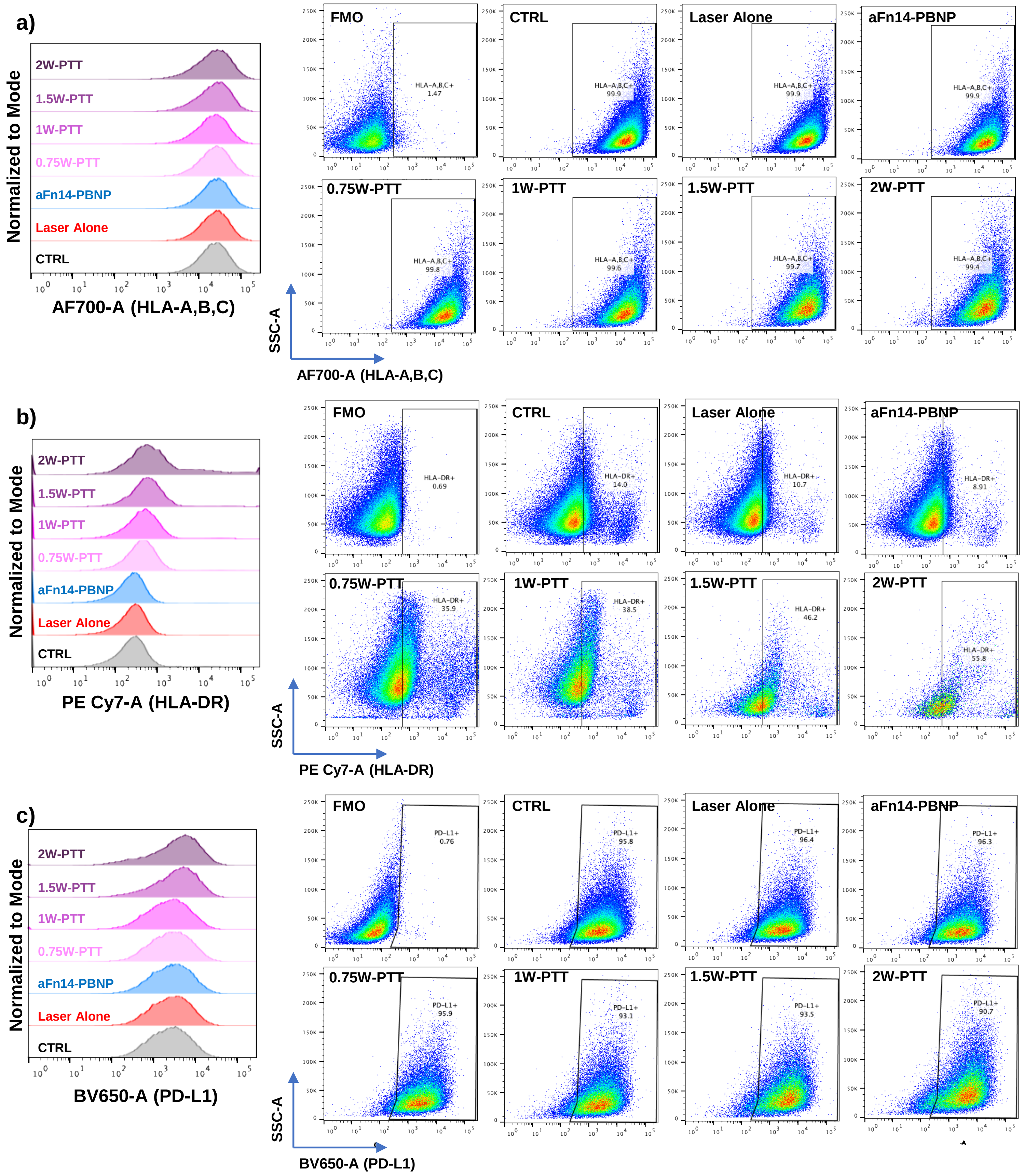

a)
Normalized to Mode
2W-PTT
1.5W-PTT
1W-PTT
0.75W-PTT
aFn14-PBNP
Laser Alone
CTRL
AF700-A (HLA-A,B,C)
FMO
HLA-A,B,C+ 1.47
CTRL
HLA-A,B,C+ 99.9
Laser Alone
HLA-A,B,C+ 99.9
aFn14-PBNP
HLA-A,B,C+ 99.9
0.75W-PTT
HLA-A,B,C+ 99.8
1W-PTT
HLA-A,B,C+ 99.6
1.5W-PTT
HLA-A,B,C+ 99.7
2W-PTT
HLA-A,B,C+ 99.4
SSC-A
AF700-A (HLA-A,B,C)
b)
Normalized to Mode
2W-PTT
1.5W-PTT
1W-PTT
0.75W-PTT
aFn14-PBNP
Laser Alone
CTRL
PE Cy7-A (HLA-DR)
FMO
HLA-DR+ 0.69
CTRL
HLA-DR+ 14.0
Laser Alone
HLA-DR+ 10.7
aFn14-PBNP
HLA-DR+ 8.91
0.75W-PTT
HLA-DR+ 35.9
1W-PTT
HLA-DR+ 38.5
1.5W-PTT
HLA-DR+ 46.2
2W-PTT
HLA-DR+ 55.8
SSC-A
PE Cy7-A (HLA-DR)
c)
Normalized to Mode
2W-PTT
1.5W-PTT
1W-PTT
0.75W-PTT
aFn14-PBNP
Laser Alone
CTRL
BV650-A (PD-L1)
FMO
PD-L1+ 0.76
CTRL
PD-L1+ 95.8
Laser Alone
PD-L1+ 96.4
aFn14-PBNP
PD-L1+ 96.3
0.75W-PTT
PD-L1+ 95.9
1W-PTT
PD-L1+ 93.1
1.5W-PTT
PD-L1+ 93.5
2W-PTT
PD-L1+ 90.7
SSC-A
BV650-A (PD-L1)

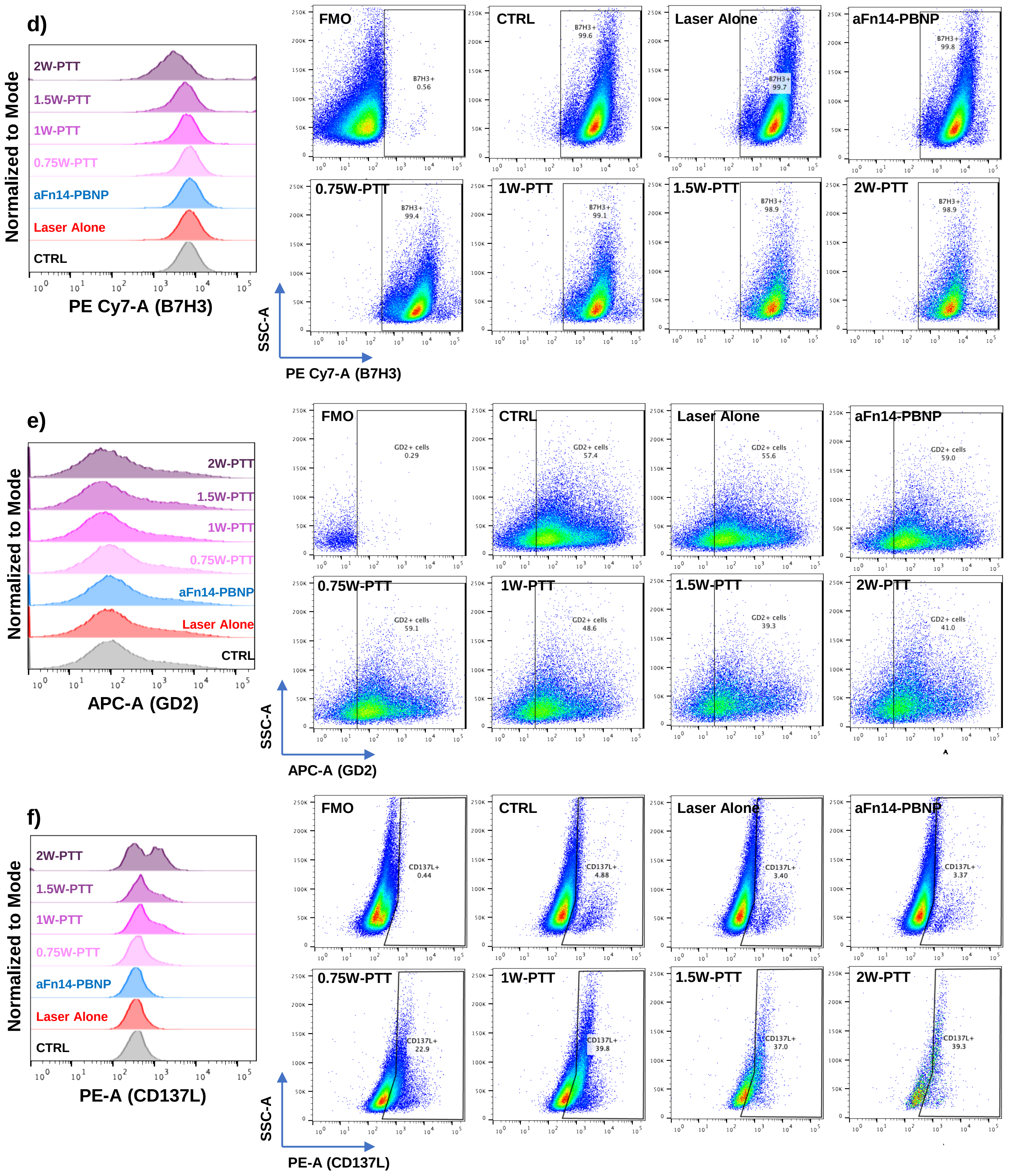

d)
Normalized to Mode
2W-PTT
1.5W-PTT
1W-PTT
0.75W-PTT
aFn14-PBNP
Laser Alone
CTRL
PE Cy7-A (B7H3)
FMO
B7H3+ 0.56
CTRL
B7H3+ 99.6
Laser Alone
B7H3+ 99.7
aFn14-PBNP
B7H3+ 99.8
0.75W-PTT
B7H3+ 99.4
1W-PTT
B7H3+ 99.1
1.5W-PTT
B7H3+ 98.9
2W-PTT
B7H3+ 98.9
SSC-A
PE Cy7-A (B7H3)
e)
Normalized to Mode
2W-PTT
1.5W-PTT
1W-PTT
0.75W-PTT
aFn14-PBNP
Laser Alone
CTRL
APC-A (GD2)
FMO
GD2+ cells 0.29
CTRL
GD2+ cells 57.4
Laser Alone
GD2+ cells 55.6
aFn14-PBNP
GD2+ cells 59.0
0.75W-PTT
GD2+ cells 59.1
1W-PTT
GD2+ cells 48.6
1.5W-PTT
GD2+ cells 39.3
2W-PTT
GD2+ cells 41.0
SSC-A
APC-A (GD2)
f)
Normalized to Mode
2W-PTT
1.5W-PTT
1W-PTT
0.75W-PTT
aFn14-PBNP
Laser Alone
CTRL
PE-A (CD137L)
FMO
CD137L+ 0.44
CTRL
CD137L+ 4.88
Laser Alone
CD137L+ 3.40
aFn14-PBNP
CD137L+ 3.37
0.75W-PTT
CD137L+ 22.9
1W-PTT
CD137L+ 39.8
1.5W-PTT
CD137L+ 37.0
2W-PTT
CD137L+ 39.3
SSC-A
PE-A (CD137L)

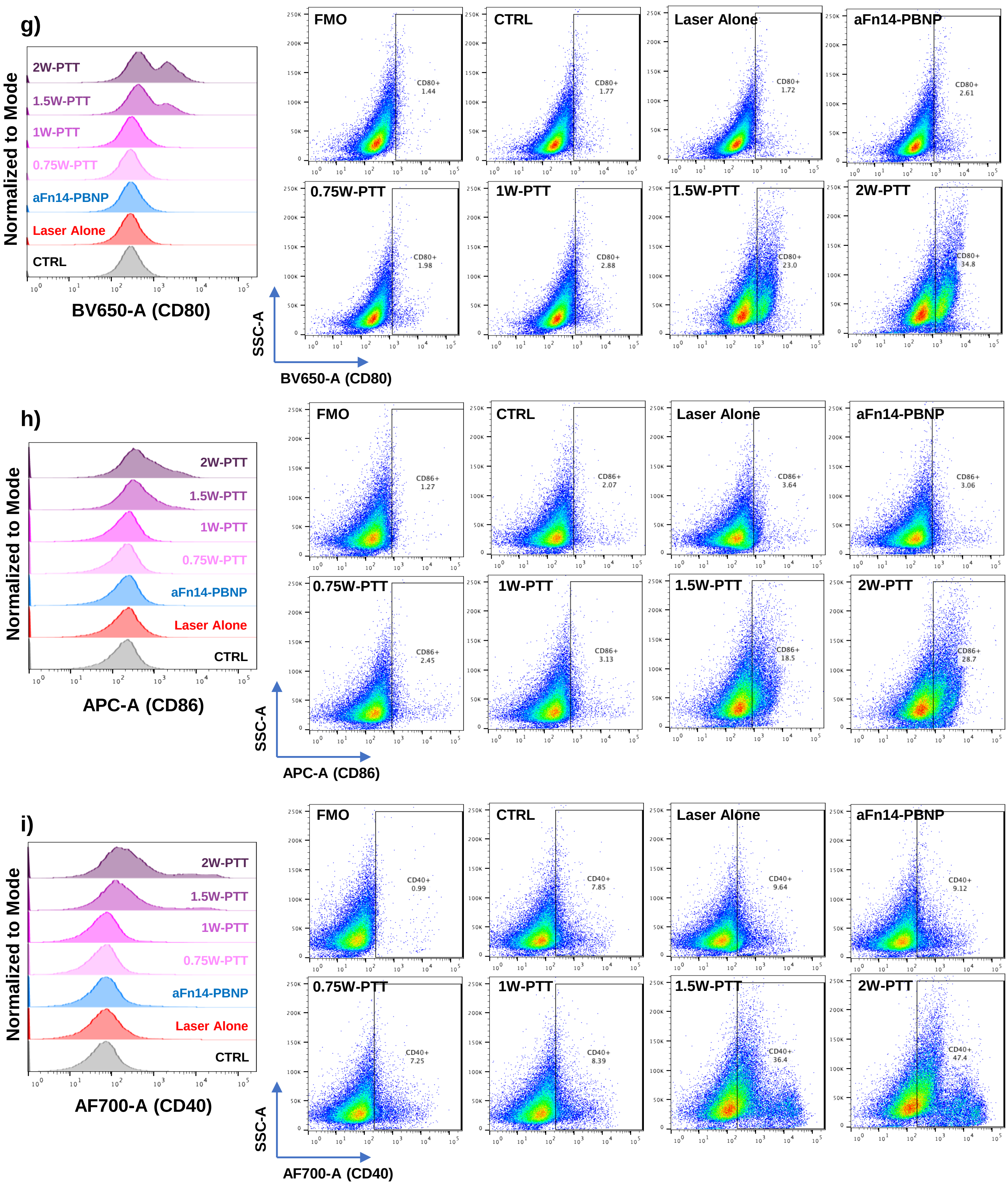

Figure S5: **Flow cytometry histograms and dot plots of expression** of (a) HLA-A,B,C, (b) HLA-DR, (c) PD-L1, (d) B7H6, (e) GD2, (f) CD137L, (g) CD80, (h) CD86, and (i) CD40 in the human U251 GBM cell line both before (CTRL) and following PTT with aFn14-PBNP at various laser powers. FMO = fluorescence minus one; CTRL = untreated cells; Laser alone = untreated cells irradiated with laser; aFn14-PBNP = cells treated with aFn14-PBNP but not irradiated; remaining conditions were treated with aFn14-PBNP and then irradiated at indicated laser power. See Figure S3A for gating strategy.

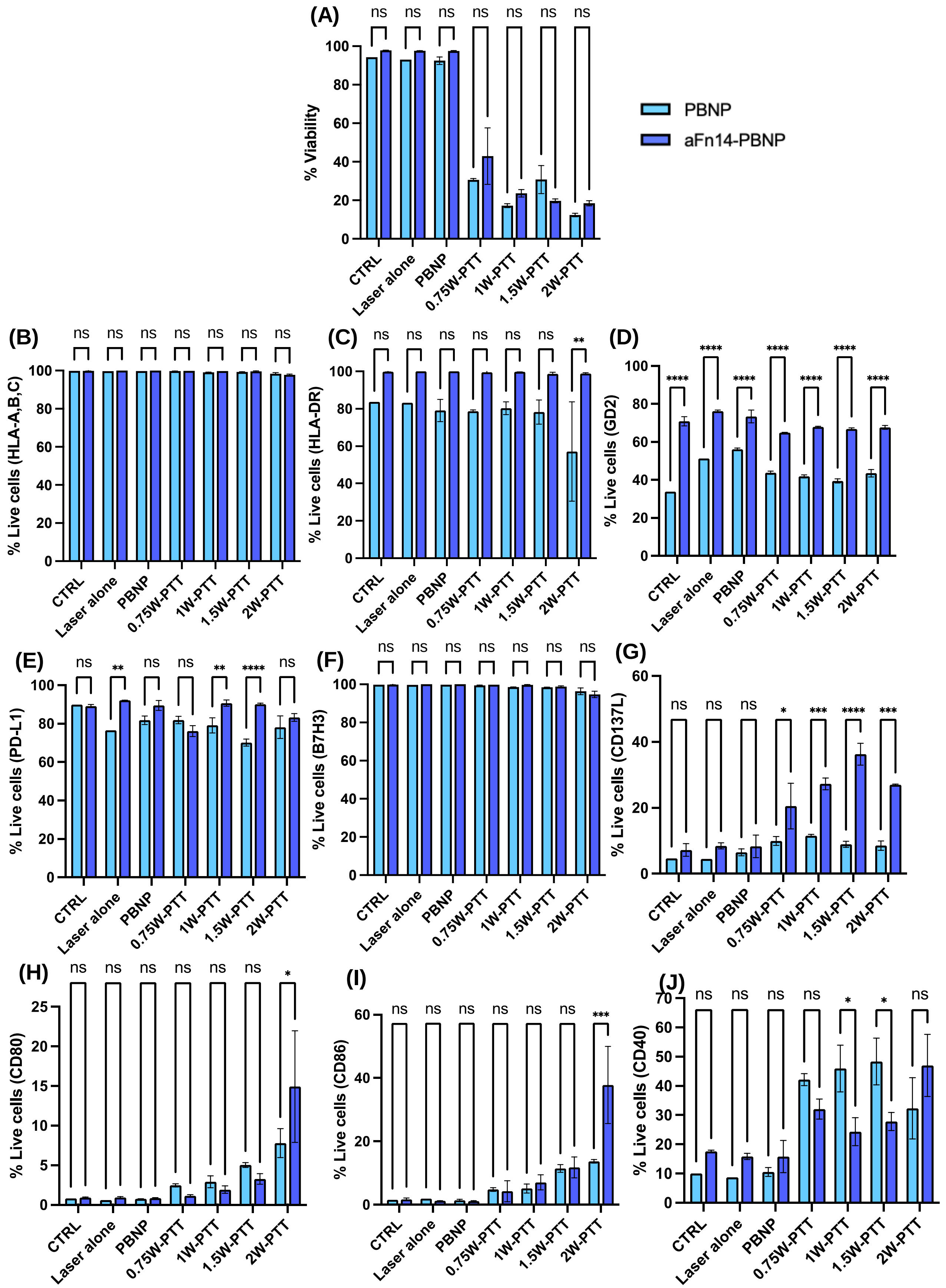

Figure S6. PTT using aFn14-PBNP triggers thermal and immunogenic cell death in a manner similar to PBNPs in U87 tumor cells. (A) U87 undergo thermal cell death after PTT with aFn14-PBNP or PBNP with increasing laser powers (0.75W-2W). **(B-J)** PTT-induced changes in immunophenotype in GBM tumor lines, including MHC expression **(B,C)**, tumor specific antigen expression (**D**), immune checkpoint inhibitor expression **(E,F),** and T cell costimulatory markers (**G,H,I,J**). CTRL = untreated cells not irradiated; laser alone = untreated cells irradiated with laser; aFn14-PBNP = aFn14-PBNP-treated cells not irradiated; remaining conditions are aFn14-PBNP-treated cells irradiated at indicated laser powers. *=$p<0.05$; **=$p<0.01$; ***=$p<0.001$; ****=$p<0.0001$.

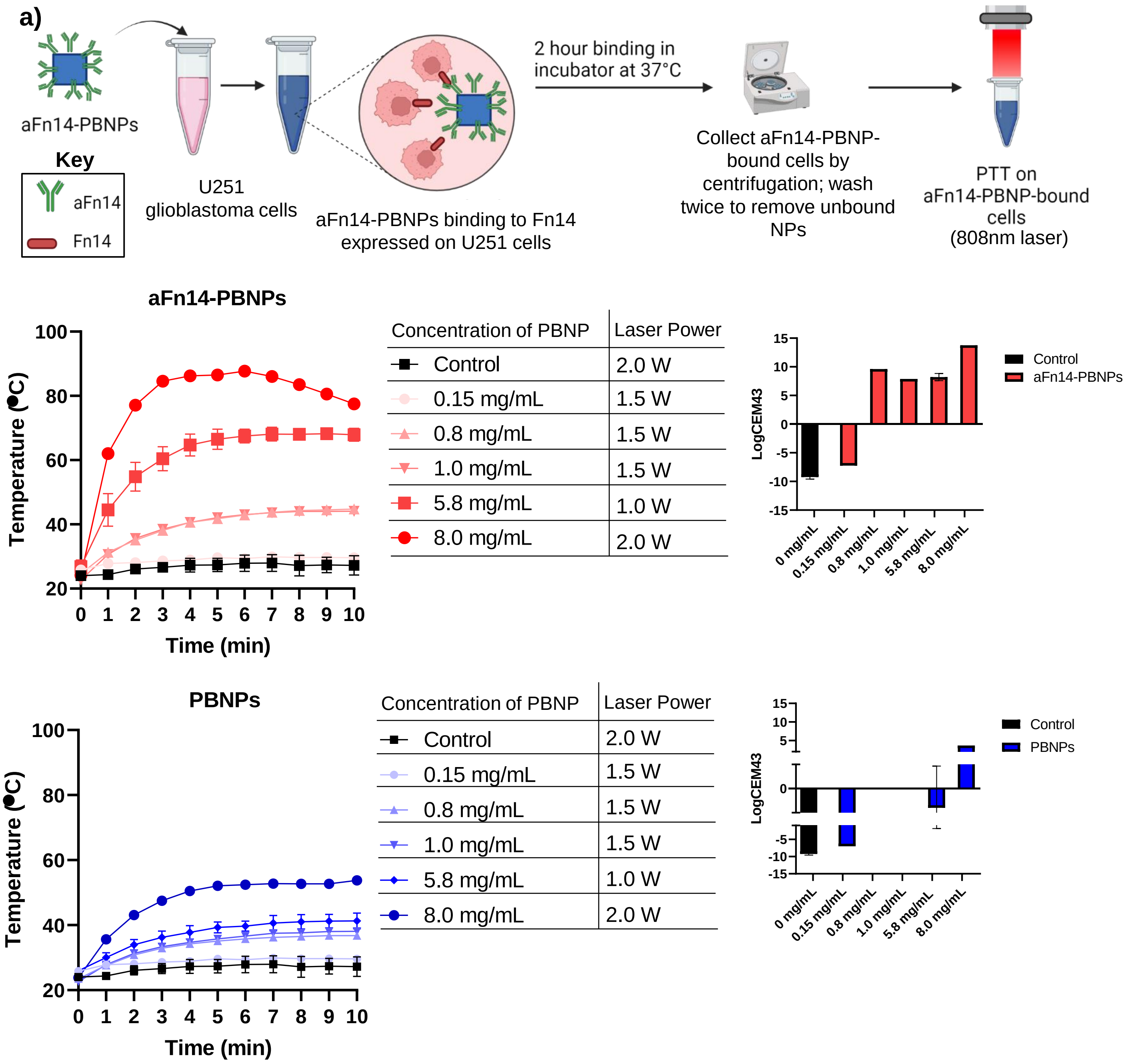

Figure S7: Determination of the concentration of aFn14-PBNP needed for the targeted PTT assay. (a) Schematic of the approach. U251 cells were incubated with varying concentrations of aFn14-PBNPs or PBNPs for 2 h at 37 °C, washed twice to remove unbound nanoparticles and/or antibody, and irradiated with a 808 nm laser for 10 min. (b-c) Heating curves (b) and the corresponding thermal doses administered (c) to U251 after incubation with various concentrations of aFn14-PBNPs during PTT. (d-e) Heating curves (d) and corresponding thermal doses administered (e) to U251 after incubation with various concentrations of PBNPs during PTT. Contacting U251 with 5.8 mg/mL aFn14-PBNP concentration yielded the best heating curves as determined by the greatest difference in heating/thermal dose between the targeted (treated with aFn14-PBNP) and untargeted (treated with PBNP) PTT conditions. Therefore, this concentration was selected for the targeted PTT studies.

STEP-BY-STEP PROTOCOLS

S2.1. Synthesis of PBNPs

Materials:

- Iron (III) chloride hexahydrate, $FeCl_3{\cdot}6H_2O$ (MW 270.3; source Sigma)
- Potassium hexacyanoferrate (II) trihydrate, $K_4[Fe(CN)_6]{\cdot}3H_2O$ (MW 422.39; source Sigma)
- Acetone (sigma)
- Citrate
- 5 M NaCl in water

Protocol:

1. Turn on two stir plates to 60C. Get them up to temperature before adding the solutions to them. Also get a third, RT plate.
2. Heat 20 mL 10.0 mM aqueous $FeCl_3$ solution under stirring to 60 ºC. This is the aqueous phase.
3. Heat 20 mL 10.0 mM $K_4[Fe(CN)_6]$ solution in water under stirring at 60 ºC. This is the organic phase.
    a. Dissolve for about 5 min and check if it dissolved. Continue until it is completely dissolved.
4. Add 961 mg Citrate to each solution. Allow it to dissolve. Add it while the solutions are stirring. Otherwise skip this step.
5. Add the $K_4[Fe(CN)_6]$ dropwise to the FeCl3 (only fill up a p1000 tip to 1 mL at a time; don't immerse the tip) - a clear dark blue dispersion will form
6. After stirring for 1min at 60C, allow the solution to cool to room temperature with the stirring continued for another 5 min at room temperature. (stir on non-heated, room-temperature plates).
7. While cooling, make ~100mL 5.0M NaCl
8. Divide the solution formed in two 50mL falcon tubes (20mL each). Try to get every drop to maximize collection yield.
9. Add an equal volume of acetone to the dispersion in each tube (in fume hood if possible)
10. Add 5mL of 5.0 M NaCl to each tube (facilitates the pellet formation) and vortex. Use a 1mL pipette and dump it in
11. Centrifuge at 10,000 rpm for ~15 min to form a pellet of Prussian Blue nanoparticles
12. While centrifuging, clean the beakers etc very soon after use with ethanol to remove the organic phase and water or PBS to remove the aqueous phase
13. Remove supernatant (hazardous waste container).
14. Combine the tubes to one single 50mL falcon from now on
15. Re-disperse the pellet in ~20 mL distilled water by sonication (amp 40%; 30 sec) and add an equal volume of acetone, mix well and add 5 mL of 5.0 M NaCl
16. Centrifuge at 10,000 rpm for 15min
17. Repeat this purification process two more times (steps 14-17). When removing the last supernatant, try to get every last drop so that the acetone doesn't cause the NPs to crash out.
18. Finally re-suspend the pellet in 10mL water and store at room temperature
19. If using for cell culture, filter-sterilize the NPs at this point through 0.22um filter
20. Measure DLS, Zeta potential, UV-Vis (characteristic peek at 700nm), Absorbance to measure the concentration from standard curve (or) dry the sample to powder and weigh to get mg/mL concentration of the prepared batch of PBNPs

S2.2. Synthesis of bioconjugated anti-Fn14-PBNP

Materials

- PBNPs (synthesized previously)
- EDC solution
- MES buffer (0.1 M MES + 0.5 M NaCl, pH = 5)
- 8.9 mg/mL 2-mercaptoethanol
- 0.1 M hydroxylamine

Protocol

1. Combine 21.5 uL of 23.21 mg/mL PBNPs with 100 uL 2.2 mg/mL EDC solution and 100 uL 8.0 mg/mL Sulfo-NHS solution in 1 mL MES buffer for 15min at RT to allow crosslinking
2. Stop the reaction by adding 100 uL 8.9 mg/mL 2-mercaptoethanol
3. Centrifuge at 22,000 xg for 30min at RT
4. Decant supernatant
5. Resuspend particles in 1 mL MES buffer
6. Sonicate using a microtip probe at 40% amplitude for 30 s to achieve a homogeneous colloidal solution
7. Add the anti-Fn14 antibody to a final concentration of 0.25 ug/mL, which corresponds to a 1:2000 mass-to-mass ratio of anti-Fn14 to PBNP
8. Allow the mixture to contact in the dark at RT for 3h on an orbital shaker
9. Add 100 uL 0.1M hydroxylamine to quench remaining primary amine sites
10. Centrifuge 22,000 xg for 30min at RT
11. Decant supernatant
    a. Save some supernatant for determining attachment efficiency (see protocol 2.3)
12. Resuspend particles in 1 mL deionized water
13. Sonicate using a microtip probe at 40% amplitude for 30 s to achieve a homogeneous colloidal solution
14. Repeat steps 10-13 twice more for a total of three washes
15. After the final spin, resuspend the particles in DI water and store them at 4C, protected from light

S2.3. Attachment efficiency of anti-Fn14 to PBNPs Materials

- Anti-Fn14-PBNP synthesis supernatants
- MES buffer
- Anti-Fn14 antibody
- Plate reader

Protocol

1. Generate a standard curve of fluorescence intensity vs known concentrations of FITC-conjugated antibody by doing two-fold serial dilutions in MES buffer: 2.0, 1.0, 0.5, 0.25, and 0.125 ug/mL antibody in water
2. Take 100uL of each of the serial dilutions and pipette into a 96well flat bottom plate
3. Take 100uL of the anti-Fn14-PBNP synthesis supernatants and add to 96well flat bottom plate
4. Read the fluorescence intensity of each well (λ_{em} = 490 nm, λ_{ex} = 525 nm) using a SpectraMax i3x Multimode Microplate Reader
5. Use the serial dilutions to generate a standard curve of fluroecence intensity vs concentration.
    a. Use this curve to determine the concentration of unbound anti-Fn14.
    b. Subtract this mass from the total mass of antibody added to the anti-Fn14-PBNP synthesis to determine the mass that remained bound to the particles.
    c. Divide the mass that remained bound by the total mass added and multiply by 100 to determine the attachment efficiency.

S2.4. Characterization of anti-Fn14-

PBNP Materials

- Zetasizer Nano ZS
- Genesys 10S spectrophotometer
- DI water
- PBNPs
- Anit-Fn14-PBNPs
- Plastic cuvette for DLS
- Plastic Zeta cuvette
- Quartz cuvette

Protocol: DLS

1. Dilute PBNPs and anti-Fn14-PBNPs in 750uL DI water in a disposable plastic cuvette for measuring DLS
2. Insert into Zetasizer and measure hydrodynamic diameter up to 1000 um using Malvern Panalytical software
3. Do this on day of synthesis, day +2, +4, +8, +16, and +20

Protocol: Zeta potential

1. Dilute PBNPs and anti-Fn14-PBNPs in 750uL DI water in a disposable plastic cuvette for measuring zeta potential using Malvern Panalytical software
2. Insert into Zetasizer and measure zeta potential
3. Do this on day of synthesis, day +2, +4, +8, +16, and +20

Protocol: UV-Vis-NIR Spectroscopy

1. Dilute PBNPs and anti-Fn14-PBNPs in 750uL DI water in a quartz cuvette for measuring absorbance using VISIONlite software
2. Insert into spectrophotometer and measure absorbance over the UV-Vis-NIR range
3. Do this on day of synthesis, day +2, +4, +8, +16, and +20

S2.5. Cell lines and culture Materials

- Cryopreserved U87 or U251
- Cell culture media: Eagle's Minimal Essential Medium containing L-glutamine + 10% fetal bovine serum + 1% penicillin/streptomycin antibiotic + 1% non-essential amino acids
- Tissue culture treated cell culture flasks
- Trypsin + EDTA
- Phosphate buffered saline (PBS)
- Luna cell counter
- Acridine orange

Protocol: Thawing cells from liquid nitrogen

1. Thaw 1 1 mL vial of cells frozen in cell culture media containing 10% DMSO into 9mL fresh culture media, prewarmed to 37C
2. Centrifuge cells at 400 xg for 5min
3. Resuspend cells in fresh culture media and plate at needed density onto tissue culture-treated flask

Protocol: Culturing cells

1. When cells are 80% confluent or when cell culture media turns yellow, passage cells as follows:
    a. Remove and save all media
    b. Wash once with PBS and add to media
    c. Add trypsin + EDTA (enough to cover the bottom of the flask). Incubate at 37C for up to 5min
    d. When all cells are lifted, remove trypsin and add to media+PBS
    e. Use fresh culture media (twice the volume of trypsin used) to wash the flask; add this media to the trypsin, media, and PBS tube
    f. Spin cells 400 xg for 5 min
    g. Aspirate supernatant
    h. Resuspend cell pellet in fresh culture media
    i. Count cells and measure viability using a Luna counter and acrylidine orange to stain for dead cells
    j. Replate at desired density or proceed to experiments

S2.6. Characterization of the PTT properties of anti-Fn14-PBNP Materials

- PBNPs and anti-Fn14-PBNPs previously synthesized
- 808 nm NIR continuous wave collimated diode laser (Laserglow Technologies)
- DI water
- Power meter (Thorlabs)
- i7 thermal imaging camera (FLIR)
- Trypsin + EDTA
- Cell culture media
- U87 cells
- U251 cells

Protocol for Figures 2B-E (PTT of PBNPs and anti-Fn14-PBNPs in water):

2. Dilute PBNPs and anti-Fn14-PBNPs to 0.15 mg/mL in DI water (mass is based on mass of PBNPs and excludes anti-Fn14) in 1.5 mL microcentrifuge tubes. Make 3 mL total of this solution for the PBNPs and for the anti-Fn14-PBNPs.
3. For each type of PBNP, aliquot 0.5 mL into each of 5 tubes: one for particles alone (no laser) and one for each of particles with 0.75, 1.0, 1.5, and 2.0 W. For clarity, there should be one tube per PBNP type (PBNP or anti-Fn14-PBNP) for each of the following conditions:
    a. No laser
    b. 0.75 W
    c. 1.0 W
    d. 1.5 W
    e. 2.0 W
4. Make a water-only blank for the laser alone condition: do not add any PBNPs or anti-Fn14-PBNPs. Only use 0.5 mL DI water.
5. Calibrate the NIR laser for PTT using a power meter. Start at 0.75W.
6. Place one PBNP + water sample directly under the laser for 10min. Use the thermal camera to measure the temperature of the water at each minute. Record these temperatures.
7. Repeat step 6 for the anti-Fn14-PBNP + water sample.
8. Repeat steps 5-6 for each sample at each remaining laser power to be tested (1.0 W, 1.5 W, 2.0 W)
9. For the PBNP and anti-FN14-PBNP alone conditions, repeat step 6 but do NOT turn on the laser.
10. For the laser alone condition, repeat step 6 using water only.
11. Calculate the thermal dose for each sample at each laser power using the CEM43°C formula, as shown in Equation (1), where t_i is the *i*-th time interval, R is related to the temperature dependence of the rate of cell death (R(T<43°C) = 0.25, R(T>43°C) = 0.5) and T is the average temperature during time interval t_i [45–47].:

$$\boldsymbol{CEM43°C} = \sum_{i=1}^{n} \boldsymbol{t_i} * \boldsymbol{R^{(43-t_i)}} \quad (1)$$

Protocol for Figure 2F (cyclic heating):

1. Dilute PBNPs and anti-Fn14-PBNPs to 0.15 mg/mL in DI water (mass is based on mass of PBNPs and excludes anti-Fn14) in 1.5 mL microcentrifuge tubes. Make 0.5 mL total for each sample in a 1.5 mL microcentrifuge tube.
2. Calibrate the NIR laser for PTT using a power meter. Start at 0.75W.
3. Place the PBNP + water sample directly under the laser for 1min. Use the thermal camera to measure the temperature of the water at each minute. Record these temperatures.
4. Turn the laser off for 10min. Use the thermal camera to measure the temperature of the water at each minute. Record these temperatures.

5. Repeat steps 3-4 two more times for a total of 3 cycles.
6. Repeat steps 3-4 three more times, this time using the anti-Fn14-PBNP sample instead of the PBNP sample.

Protocol for Figure 2G-J (PTT of PBNPs and anti-Fn14-PBNPs in U87 or U251 cell suspensions):

1. Harvest the U87 and U251 cells as described in Protocol 2.5, Culturing Cells.
2. Resuspend cells with PBNPs or anti-Fn14-PBNPs in cell culture media such that each 0.5 mL of media contains 5e6 cells + 0.15 mg/mL PBNP. Make a total of 3 mL per condition. For clarity, the conditions are below:
    a. 5e6 U87 + 0.15 mg/mL PBNP in 0.5mL culture media
    b. 5e6 U87 + 0.15 mg/mL anti-Fn14 PBNP in 0.5mL culture media
    c. 5e6 U251 + 0.15 mg/mL PBNP in 0.5mL culture media
    d. 5e6 U251 + 0.15 mg/mL anti-Fn14-BNP in 0.5mL culture media
3. Aliquot 0.5 mL of each condition above into separate 1.5 mL microcentrifuge tubes. Repeat until there are 5 tubes per condition. Each tube will get one of the following treatments:
    a. No laser
    b. 0.75 W
    c. 1.0 W
    d. 1.5 W
    e. 2.0 W
4. Make a laser only control, which is 0.5 mL DI water
5. Calibrate the NIR laser for PTT using a power meter. Start at 0.75W.
6. For each sample EXCEPT for the no laser controls, place the sample directly under the laser for 1min. Use the thermal camera to measure the temperature of the water at each minute. Record these temperatures.
7. For the no laser controls, repeat step 5 but do not turn on the laser.
8. SAVE all of the cells for use in further analysis (see Protocol 2.7).

S2.7. Elucidation of the glioblastoma tumor cell phenotype post-PTT Materials:

1. Cell culture media
2. U87 and U251 PTT-treated cells from Protocol 2.7
3. TrypLE
4. 6 well tissue culture-treated plates
5. CellTiter-Glo Luminescent Viability Assay
6. i3 SpectraMax
7. Flow cytometry reagents:
   - Zombie Violet™ fixable viability dye
   - Flow buffer (PBS + 1% FBS)
   - Fc Block : BioLegend® (TruStain fcX™ (anti-mouse CD16/32)
   - Flow buffer (PBS + 1%FBS)
   - BD Cytofix/Cytoperm™ : BD Bioscience 51-2090KZ
   - BD Perm/Wash™ : BD Bioscience 51-2090KZ
   - Zombie Violet™ Fixable Viability Kit (BioLegend #423114)
   - ICD Panel 1:
     - APC anti-human GD2 Antibody (BioLegend #357306) – volume needed: 125uL
     - PE anti-human CD137L Antibody (Biolegend #311504) – volume needed: 125uL
     - AlexaFluor 700 anti-human HLA-A,B,C Antibody (Biolegend #311438) – volume needed: 125uL
     - PE/cy7 anti-human B7H3 Antibody (Biolegend #351008) – volume needed: 125uL
     - Brilliant Violet 650 anti-human PD-L1 Antibody (Biolegend #329740) – volume needed: 125uL
   - ICD Panel 2:
     - PE anti-human Fn14 Antibody (BioLegend #314004) – volume needed: 62.5uL
     - APC anti-human CD86 Antibody (Biolegend #374208) – volume needed: 125uL
     - Brilliant Violet 650 anti-human CD80 Antibody (Biolegend #305227) – volume needed: 125uL
     - Alexa Fluor® 700 anti-human CD40 Antibody (Biolegend #334327/334328) – volume needed: 125uL
     - PE/cy7 anti-human HLA-DR Antibody (Biolegend #307615) – volume needed: 125uL
   - ICD Panel 3:
     - HMGB1-AlexaFluor 647 : ab195011 (Rb mAb to HMGB1: 100uL volume; 0.5mg/mL)
     - Calreticulin PE : ab209577 (Rb mAb to Calreticulin; 100uL; 0.5mg/mL)
   - ICD Panel 4:
     - Isotype control: ab209478: Rabbit IgG, monoclonal Isotype control PE (100uL; 0.5ug/mL)

Protocol for preparation of cells for ATP assay and ICD staining:

1. Harvest the U87 and U251 cells from Protocol 2.6, Protocol for Figure 2G-J.
2. Centrifuge the cells at 400 xg for 5min at RT
3. Resuspend the cells in their respective cell culture media and plate them in 6 well plates at 37 °C for 24 h at 1e6 cells/mL
4. Harvest the cell culture media and the cells from the plates using TrypLE and transfer to 15 mL conical tubes
5. Centrifuge the cells at 400 xg for 5 min
6. Aspirate supernatants
7. Resuspend the cell pellets in PBS for further ATP assay and ICD analysis (below) at 1e6 cells per 100uL PBS.

Protocol for ATP assay using the CellTiter-Glo Luminescent Viability Assay (this follows the manufacturer's protocol):

1. Aliquot 100 uL of cells in PBS into 96-well opaque bottom plate.
    a. Include a well with 100 uL PBS without cells as a control.
2. Bring ATP assay reagents to at RT and mix them together as indicated by manufacturer
3. Aliquot the ATP reagent onto the cells (100 µL per well)
4. Cover the plate in foil and place on an orbital shaker for 2 min.
5. Incubate the plate at RT for 5 to 10 min
6. Measure luminescence via SpectraMax.

Protocol for flow cytometry analysis of ICD markers:

1. Label Flow tubes in duplicates
    a. Panel 1 (GD2; CD137L; HLA-A,B,C; B7H3; PD-L1)
    b. Panel 2 (Fn14; CD80; CD86; CD40; HLA-DR)
    c. Panel 3 (ICD: Calreticulin and HMGB-1)
    d. Panel 4 (Isotype: Calreticulin Isotype)
2. Add 100uL of cell suspension in PBS to each panel
    a. There are 4 panels, thus use up 400uL of the cell suspension
    b. Store the rest of the cell suspension in the falcon tube (this will be used for ATP assay later)
3. Add 3mL PBS to all flow tubes (4 panels in duplicates) and spin at 1400 rpm/400g for 5min
4. Discard supernatant and wash again with PBS
5. Discard supernatants for all tubes, add 1uL Zombie violet viability dye (reconstituted at the manufacturer's recommended concentration) to all the tubes (all four panels and FMOs) apart from unstained group
6. Incubate at RT, covered in Aluminum foil for 20min
7. During this incubation time, prepare the Antibody cocktails for panels 1 and 2:
    a. Panel 1:
        i. APC-GD2: 5uL/tube
        ii. PE-CD137L: 5uL/tube
        iii. AF700-HLA-A,B,C: 5uL/tube
        iv. PE/Cy7 B7H3: 5uL/tube
        v. BV650 PD-L1: 5uL/tube
        vi. Total number of samples to be stained: 20 samples (10 samples in duplicate). Prepare the cocktail for 21 samples. Therefore in an Eppendorf add 105uL GD2 + 105uL CD137L + 105uL HLA-A,B,C + 105uL B7H3 + 105uL PD-L1 antibodies.
        vii. Add 25uL per tube in panel 1
    b. Panel 2:
        i. PE-Fn14: 2.5uL/tube
            1. <=0.5ug/million in 100uL (stock conc: 0.2mg/mL)
        ii. APC CD86: 5uL/tube
        iii. BV650 CD80: 5uL/tube
        iv. AF700 CD40: 5ul/tube
        v. PE/Cy7-HLA-DR: 5uL/tube

vi. number of samples to be stained: 20 samples (10 samples in duplicate). Prepare the cocktail for 21 samples. Therefore, in an Eppendorf add 52.5uL Fn14 + 105uL CD86 + 105uL CD80 + 105uL CD40 + 105ul HL-DR antibodies.
vii. Add 22.5uL per sample

8. After 20min incubation, wash with 2-3mL flow buffer at 400g, 5min
9. Discard supernatant and wash again with 2mL flow buffer
10. Discard supernatant and block all samples by adding 5 uL blocking reagent (Fc Block) to all tubes
11. Incubate at 4ºC for 10 minutes
    a. Note: No need to wash after blocking, you can add the surface stain directly after this 10min blocking step
12. Stain for Calreticulin and Isotype in panel 3 and 4
    a. For Panel 3
        i. For 20 tubes, prepare PE-calreticulin for about 25 samples
        ii. Add 12.5uL PE-calreticulin antibody with 12.5uL PBS
        iii. Add 1uL/tube, in all tubes in panel 3 except unstained
    b. For Panel 4
        i. For 20 tubes, prepare PE-Isotype for about 25 samples
        ii. Add 12.5uL PE-Isotype antibody with 12.5uL PBS
        iii. Add 1uL/tube, in all tubes in panel 3 except unstained
    c. Incubate all tubes at 4deg for 30min
13. Start the surface staining for Panel 1 and 2 and FMOs
    a. Panel 1
        i. Add 25uL of Antibody cocktail 1 to each sample for panel 1 except unstained and FMOs of that panel
    b. Panel 2
        i. Add 22.5uL of Antibody cocktail 2 to each sample for panel 2 except unstained and FMOs of that panel
    c. FMO controls (untreated cells with all antibodies except the one read)
        i. Add same amount of antibody used for staining the samples
        ii. Panel 1:
            1. FMO 1 (APC GD2) → add 5uL of CD137L, HLA-A,B,C, B7H3 and PD-L1
            2. FMO 2 (PE CD137L)→ add 5uL of GD2, HLA-A,B,C, B7H3 and PD-L1
            3. FMO 3 (AF700 HLA A, B, C)→ add 5uL of GD2, CD137L, B7H3 and PD-L1
            4. FMO 4 (PE/Cy7 B7H3)→ add 5uL of GD2, CD137L, HLA-A,B,C, and PD-L1
            5. FMO 5 (BV650 PD-L1)→ add 5uL of GD2, CD137L, HLA-A,B,C and B7H3
        iii. Panel 2:
            1. FMO 6 (PE Fn14)→ add 5uL of CD86, CD80, CD40 and HLA-DR
            2. FMO 7 (APC CD86)→ add 2.5uL of Fn14, 5uL of CD80, CD40 and HLA-DR
            3. FMO 8 (BV650 CD80)→ add 2.5uL of Fn14, 5uL of CD86, CD40 and HLA-DR
            4. FMO 9 (AF700 CD40)→ add 2.5uL of Fn14, 5uL of CD80, CD86 and HLA-DR
            5. FMO 10 (PE/Cy7 HLA-DR)→ add 2.5uL of Fn14, 5uL of CD80, CD86 and CD40
14. Incubate all panels and FMO controls for 20min at 4 degree

15. Wash all tubes (all four panels after their respective incubation time) in 3mL Flow buffer (400g spin for 5min)
16. Wash Panel 1 and panel 2 and FMOs again in flow buffer, discard supernatant
17. Resuspend panel 1, 2 and FMOs in 200uL Flow buffer, cover in Aluminum foil and store at 4deg for flow cytometry analysis
18. Fix and permeabilize the cells in Panel 3 and 4 (ICD and isotype panel)
19. Re-suspend cell pellet of both panels in 250uL cytofix/cytoperm (this buffer is a part of a kit, it is stored in 4deg fridge under the centrifuge)
20. Incubate at 4ºC for 20 minutes
21. Prepare Perm/wash buffer. This comes as 10x solution; prepare 50mL of 1x solution. Combine 5mL Perm/wash buffer + 45mL of Flow buffer
22. After 20min incubation, add 250uL 1x perm/wash in each tube
23. Spin at 1000 x g for 5 minutes; discard supernatant
24. Add 100µL perm/wash to all tubes
25. HMGB-1 staining: add 2uL AF647-HMGB1 antibody to panel 3 alone
26. Incubate both panel 3 and 4 at 4ºC for 30 minutes
27. Prepare compensation beads during this 30min incubation:
    a. Label Flow tubes
        i. Zombie violet (viability dye, use arc amine beads for this tube)
        ii. PE comp
        iii. APC comp
        iv. BV650 comp
        v. AF700 comp
        vi. PE/Cy7 comp
    b. Add 1 drop of arc amine (reactive bead, green cap) in zombie violet tube alone
    c. Add 1drop of ultra comp beads to all other tubes
    d. Add 1uL of zombie violet viability dye to "zombie violet" tube
    e. Add 1uL of any PE antibody to PE tube
    f. Add 1uL of any APC antibody to APC tube
    g. Add 1uL of any BV650 antibody to BV650 tube
    h. Add 1uL of any AF700 antibody to AF700 tube
    i. Add 1uL of any PE/cy7 antibody to PE/cy7 tube
    j. Incubate all tubes at RT for 30min covered in foil
    k. Add 4mL of PBS to all tubes
    l. Spin down at 400g for 5min
    m. Discard supernatant
    n. Add 1 drop of negative bead (white capped tube) only to "zombie violet" tube. Do not add this to other tubes
    o. Add 100uL Flow buffer to all tubes and store at 4deg
    p. Add 1 mL perm/wash to all tubes to wash off excess antibody
    q. Centrifuge for 5 minutes at 1400 RPM
    r. Discard supernatant

s. Re-suspend in 200uL Flow buffer

t. Store at 4ºC coved in Al foil until flow cytometry analysis

S2.8. Determining anti-Fn14 binding to U87 and U251 cells via flow cytometry Materials:

- U87 cells
- U251 cells
- Fn14 antibody used for anti-Fn14-PBNP synthesis (FITC, clone ITEM-4, Santa Cruz Biotechnology Inc, Dalles, TX, USA)
- Fn14 antibody used for staining in the ICD panel (PE, clone ITEM-1, Biolegend)
- Flow buffer (PBS + 1% FBS)

Protocol:

1. Harvest U87 and U251 cells as described in Protocol 2.5, Culturing Cells.
2. Wash cells twice in flow buffer
3. Resuspend cells at 100,000 cells per 100 uL flow buffer and aliquot 100 uL per well into a 96 well round bottom plate. Aliquot three wells per cell line.
4. Add the manufacturer's recommended amount of antibody: add the FITC antibody to one well of each cell line and the PE antibody to another well for each cell line. Leave the third well without antibody as a negative control.
5. Incubate 25 min at 4 C in the dark
6. Wash all wells twice with 200 uL flow buffer
7. Resuspend all cells in 100 uL flow buffer and collect 50,000 events per well.
8. Use FlowJo to analyze data: gate on tumor cells, then single cells, then percent PE or FITC+

S2.9. Quantifying the attachment of anti-Fn14-PBNP to U87 tumor cells Materials:

- U87 cells
- PBNPs and anti-Fn14-PBNPs
- PBS
- trypLE
- Cell culture media
- Luna cell counter
- Acridine orange
- Teflon beakers
- High purity concentrated nitric acid
- 40um cell strianer

Protocol:

1. Plate U87 cells into 6well plates (1e6 cells per well). Do 10 wells per cell line.
2. Add PBNP to 5 wells of cells at a final concentration of 0.15 mg/mL
3. Incubate for 15, 30, 45, 60, or 120 min (one timepoint per well)
4. Repeat steps 2-3 for anti-Fn14-PBNPs (concentration based on mass of PBNP only)
5. Rinse cells with PBS
6. Harvest cells with TrypLE
7. Count cells using a Luna cell counter and acridine orange
8. Resuspend all cells in 1 mL PBS
9. Transfer cells to thick screw-top Teflon beakers and treat with high-purity concentrated nitric acid for 20 min at RT.
10. Dilute the acid to a concentration of 2% (v/v) using DI water
11. Allow digestion to continue overnight at 60 °C
12. Filter the samples through a 40 um cell strainer
13. Transfer samples to a sealed tube
14. Transport samples to the University of Maryland Plasma Mass Spectroscopy Lab. Probe for the ^{57}Fe elemental isotope of non-radioactive iron, which is a component of the PBNPs, at the core facility.

S2.10. Assessing the efficacy of using anti-Fn14-PBNP for targeted PTT of glioblastoma tumor cells Materials:

- U87 cells
- U251 cells
- PBNPs and anti-Fn14-PBNPs previously synthesized
- 808 nm NIR continuous wave collimated diode laser (Laserglow Technologies)
- Power meter (Thorlabs)
- i7 thermal imaging camera (FLIR)
- Cell culture media

Protocol:

1. Incubate 1e6 U87 or U251 cells with 5.8 mg/mL PBNP, 5.8 mg/mL anti-Fn14-PBNP, 3 μg/mL FITC-conjugated anti-Fn14 (to match the concentration of anti-Fn14 used in the anti-Fn14-PBNP condition), or a vehicle control (water) for 2 h at 37 °C in 1.5 mL microcentrifuge tubes
2. Wash cells twice to remove unbound particles and/or antibody
3. Resuspend cells in 500uL media
4. Take 50,000 cells from each condition to assess FITC expression via flow cytometry: dilute cells to 200uL in flow buffer in a 96 well round-bottom plate and collect the entire well
5. PTT-treat all remaining samples:
    a. Calibrate the NIR laser for PTT using a power meter. Start at 0.75W.
    b. For each sample, place the sample directly under the laser for 1min. Use the thermal camera to measure the temperature of the water at each minute. Record these temperatures.
6. Plate all cells into one well of a 6well plate per sample. Dilute to 2mL in cell culture media and rest overnight
7. Assess viability after 24h using the Luna cell counter and acridine orange.
